# Rescue from galactose-induced death of Leigh Syndrome patient cells by pyruvate and NAD^+^

**DOI:** 10.1038/s41419-018-1179-4

**Published:** 2018-11-14

**Authors:** Eligio F. Iannetti, Jan A. M. Smeitink, Peter H. G. M. Willems, Julien Beyrath, Werner J. H. Koopman

**Affiliations:** 1 Khondrion BV, Nijmegen, The Netherlands; 2 Department of Pediatrics, Radboud Center for Mitochondrial Medicine, Radboudumc, Nijmegen, The Netherlands; 3 Department of Biochemistry, Radboud Institute for Molecular Life Sciences, Radboud Center for Mitochondrial Medicine, Radboudumc, Nijmegen, The Netherlands

## Abstract

Cell models of mitochondrial complex I (CI) deficiency display activation of glycolysis to compensate for the loss in mitochondrial ATP production. This adaptation can mask other relevant deficiency-induced aberrations in cell physiology. Here we investigated the viability, mitochondrial morphofunction, ROS levels and ATP homeostasis of primary skin fibroblasts from Leigh Syndrome (LS) patients with isolated CI deficiency. These cell lines harbored mutations in nuclear DNA (nDNA)-encoded CI genes (*NDUFS7*, *NDUFS8*, *NDUFV1*) and, to prevent glycolysis upregulation, were cultured in a pyruvate-free medium in which glucose was replaced by galactose. Following optimization of the cell culture protocol, LS fibroblasts died in the galactose medium, whereas control cells did not. LS cell death was dose-dependently inhibited by pyruvate, malate, oxaloacetate, α-ketoglutarate, aspartate, and exogenous NAD^+^ (eNAD), but not by lactate, succinate, α-ketobutyrate, and uridine. Pyruvate and eNAD increased the cellular NAD^+^ content in galactose-treated LS cells to a different extent and co-incubation studies revealed that pyruvate-induced rescue was not primarily mediated by NAD^+^. Functionally, in LS cells glucose-by-galactose replacement increased mitochondrial fragmentation and mass, depolarized the mitochondrial membrane potential (Δψ), increased H_2_DCFDA-oxidizing ROS levels, increased mitochondrial ATP generation, and reduced the total cellular ATP content. These aberrations were differentially rescued by pyruvate and eNAD, supporting the conclusion that these compounds rescue galactose-induced LS cell death via different mechanisms. These findings establish a cell-based strategy for intervention testing and enhance our understanding of CI deficiency pathophysiology.

## Introduction

Oxidative phosphorylation (OXPHOS) is carried out by five protein complexes (CI-CV) and constitutes a prime source of mitochondrial ATP^1^. OXPHOS dysfunction and isolated CI deficiency (OMIM 252010) is often associated with Leigh Syndrome (LS), a rare metabolic disorder with poorly understood pathophysiology^2–6^. Functionally, CI couples the transfer of electrons from NADH to oxidized ubiquinone-10 to the transport of H^+^ from the mitochondrial matrix across the mitochondrial inner membrane (MIM). This process provides the electron transport chain (ETC) with reducing equivalents to sustain a trans-MIM pH gradient (ΔpH) and electrical potential (Δψ). Moreover, CI action allows operation of the tricarboxylic acid (TCA) cycle by NADH-to-NAD^+^ recycling^7–10^. We previously demonstrated that primary skin fibroblasts of pediatric LS patients exhibit disturbed calcium/ATP homeostasis^11^, partial Δψ depolarization^12^, altered mitochondrial morphology^13^, and increased reactive oxygen species (ROS) levels^14^. However, we neither detected increased lipid peroxidation nor alterations in thiol redox state in LS cells^14^. It was hypothesized that mitochondrial (CI-mediated) NADH recycling is required to prevent “reductive stress” (as opposed to “oxidative stress”) and allow cell proliferation^9^. Various CI deficiency models display an apparent reduction in NAD^+^/NADH ratio^14–16^, compatible with the key role of CI in oxidizing NADH to NAD^+^^17^ and supporting the reductive stress hypothesis. In primary skin fibroblasts, the free cytosolic NAD^+^/NADH ratio is coupled to the lactate/pyruvate (L/P) ratio^8,18^. Compatible with the reductive stress idea, patients with CI deficiency displayed a mild/severe lactic acidemia paralleled by an increased L/P ratio in the corresponding skin fibroblasts^19^. In CI-inhibited human skin fibroblasts and other cell models of CI deficiency a parallel increase in lactate production and glycolytic flux was demonstrated^15,20–23^. Although the latter is important to sustain cell viability, it might prevent triggering or interfere with the detection of other relevant consequences of CI deficiency^24,25^. Here we studied the cellular effects of genetic CI deficiency by comparing control and LS patient primary skin fibroblasts cultured in (pyruvate-free) glucose-containing or galactose-containing media. The latter enters glycolysis via the Leloir pathway at a much slower rate than glucose, preventing ATP production via rapid glucose-to-pyruvate conversion^26,27^. We demonstrate that glucose-by-galactose replacement specifically induces the death of LS fibroblasts, and that this dead is rescued by pyruvate and exogenous NAD^+^ (eNAD) via different mechanisms.

## Materials And methods

### Cell lines

Fibroblasts were obtained following informed parental consent and according to the relevant Institutional Review Boards from skin biopsies of healthy individuals and various Leigh Syndrome (LS) patients with isolated complex I (CI) deficiency (OMIM 252010). These cell lines were previously fully characterized at the genetic, biochemical and cellular level in our group (reviewed in ref. ^39^). Control cell lines included: #CT5120 (“CT1”), #CT5119 (“CT2”), and #CT5118 (“CT3”), which all displayed a similar and normal phenotype during their previous characterization (reviewed in ref. ^39^). Patient cell lines harbored mutations in various genes encoding CI structural subunits: #P5175 (“S7”, *NDUFS7*-V112M mutation), #P6603 (“S8”, *NDUFS8*-R94C mutation), and #P5866 (“V1”, *NDUFV1*-R59X/T423M mutations). S8 cells display a very low residual CI activity (i.e., 18% of the lowest control value) whereas this activity is higher for S7 (68%) and V1 (64%) fibroblasts^13^. Fibroblasts were cultured in Medium 199 (M199; #22340–020; Thermo Fisher Scientific, Waltham, MA, USA) in a humidified atmosphere (95 air, 5% CO_2_) at 37 °C. This medium contained no pyruvate, 25 mM HEPES, 5.5 mM d-glucose, 0.7 mM l-glutamine, and 0.05 mM phenol red. To this medium was added: 10% (v/v) Fetal Bovine Serum (FBS; #758093; Greiner Bio-One, Kremsmünster, Austria), 100 IU/ml penicillin/streptomycin (#30–002-CI; Corning, NY, USA). Cells were passaged every 4–5 days when reaching 90% confluence by trypsinization (#15400054; Corning) and were mycoplasma negative (Mycoalert^®^ mycoplasma detection kit; Lonza, Allendale, NJ, USA).

### Preparation of glucose and galactose media

As a starting point we used DMEM without glucose, pyruvate, glutamine and phenol red (#A1443001; Thermo Fisher). From this DMEM a “glucose medium” was prepared by adding 5.5 mM glucose (#G8270; Sigma-Aldrich, St. Louis, MO, USA). Similarly, a “galactose medium” was prepared by adding 5.5 mM galactose (#G0750, Sigma-Aldrich). To both the glucose/galactose media was added: 1 mM L-glutamine (GlutaMAX^®^; #35050061; Thermo Fisher), 10% (v/v) dialyzed Fetal Bovine Serum (FBS; #26400044; Thermo Fisher), 10 mM 4-(2-hydroxyethyl)-1-piperazineethanesulfonic acid (HEPES; #15630080; Thermo Fisher), 100 IU/ml penicillin/streptomycin (#30–002-CI; Corning), and 0.05 mM Phenol Red (#3532; Sigma-Aldrich). Glucose and galactose media were stored at 4 °C in the dark for a maximum of 1 month.

### Medium additions

In certain experiments (one of) the following compounds was/were added to the medium: sodium pyruvate (#11360070, Life Technologies, Carlsbad, CA, USA), l-aspartic acid (#A7219, Sigma-Aldrich), 2-ketobutyric acid (#K401, Sigma-Aldrich), oxaloacetic acid (#O4126, Sigma-Aldrich), sodium l-lactate (#L7022, Sigma-Aldrich), l-(-)-malic acid (#M1000, Sigma-Aldrich), sodium succinate dibasic hexahydrate (#S9637, Sigma-Aldrich), dimethyl α-ketoglutarate (#349631, Sigma-Aldrich), uridine (#U3003, Sigma-Aldrich), β-nicotinamide adenine dinucleotide hydrate (#43410, Sigma-Aldrich), Oleamide (#O2136, Sigma-Aldrich) These compounds were dissolved directly in the medium after which its pH was adjusted to 7.2 with NaOH. Media were freshly prepared prior to each experiment.

### Cell functional and viability analysis

Cells were suspended in M199 culture medium, seeded in black 96-well plates (#655090, Greiner Bio-one) at a density of 2500 cells/well, and incubated overnight in a humidified atmosphere (95 air, 5% CO_2_) at 37 °C. On the next day, M199 culture medium was removed, cells were washed twice during 1 min with PBS and glucose or galactose medium was added. Then, the cells were cultured in these media and analyzed at 24 h (functional analysis) and 96 h (cell viability analysis).

### Live-cell high-content microscopy

Fluorescence microscopy images were acquired from 96-well plates using a BD Pathway 855^®^ High-Content Bioimager (Becton Dickinson, Franklin Lakes, NJ, USA) using a protocol described previously^28^. For viability analysis, cells were stained with (a combination of) Calcein-AM (#65–0853–39; Thermo Fisher) and Hoechst 33258 (#94406, Invitrogen). Calcein-AM is membrane-permeable and accumulates in the cytosol upon esterase-mediated cleavage of its AM (acetoxymethyl) ester tail. Hoechst 33258 is also membrane-permeable and binds to the AT base pairs in the minor groove of double-stranded DNA, which greatly increases its fluorescence signal. For integrated analysis of cellular and mitochondrial morphological/functional parameters (“morphofunction”; Table S1), cells were co-stained with Calcein-AM, Hoechst 33258, and TMRM (Tetramethyl rhodamine methyl ester, #T668, Invitrogen). The latter is a fluorescent lipophilic cation that accumulates in the mitochondrial matrix depending on the magnitude of the mitochondrial membrane potential (Δψ). Importantly, under our experimental conditions TMRM operated in non-quenching mode and was present in the medium during image acquisition^28^. Calcein, Hoechst, and TMRM images were quantified as described in detail previously^28^.

### Quantification of total cellular ATP and NAD^+^ content

Using a ×10 objective (UPlanSApo, NA 0.4, Olympus, Leiderdorp, The Netherlands), 96-well plates were imaged and the total number of Calcein-positive pixels (“Casum”) was determined from the center of each well (2×2 image montage; 4 images in total) by live-cell high-content microscopy (see above). Next, the total cellular ATP content was determined using the CellTiter-Glo^®^ Luminescence Cell Viability Assay according to the manufacturer’s protocol (#G7570, Promega Corporation, Madison, WI, USA). Luminescence signals were quantified using a scanning microplate reader (FLUOstar Omega^®^, BMG Labtech, Cary, NC, USA). This signal was divided by Casum for each well to normalize for the total cell volume in each well. The latter is possible given the flat morphology of the fibroblasts (i.e., their dimensions in the XY direction greatly exceed their height in the Z-direction;^29^). In certain experiments, cells were pre-incubated for 20 min with 10 µM of Oligomycin A (#O4876, Sigma-Aldrich) prior to ATP quantification^20^. Total cellular NAD^+^ content was determined using the NAD/NADH-Glo^®^ Luminescence Assay (#G9071, Promega) according to the manufacturer’s protocol and quantified as described for the ATP measurement.

### Reactive oxygen species measurement

The levels of cellular reactive oxygen species (ROS) were measured in 96-well plates by quantifying the oxidation of two ROS reporter molecules:^30–32^ 5-(and-6)-chloromethyl-2′,7′-dichlorodihydrofluorescein diacetate (CM-H_2_DCFDA, #C6827, Thermo Fisher) and Hydroethidine (HEt, #D11347, Thermo Fisher). To this end, the cells were stained in DMEM medium (#A1443001; Thermo Fisher), which was supplemented with 10 mM HEPES (#15630080; Thermo Fisher) and contained either CM-H_2_DCFDA (1 µM) or HEt (10 µM) for 30 min (37 °C, 5% CO_2_) in the dark. After this incubation, cells were washed twice with and placed in Dulbecco’s phosphate-buffered saline (DPBS containing calcium and magnesium, #14040133, Thermo Fisher). Next, fluorescence signals were visualized using the high-content microscopy system described above (2×2 image montage; 4 images in total) using a x20 objective (UApo/340, NA 0.75, Olympus). Following acquisition, each image (RAW) was processed using the rolling ball algorithm^28^ with a feature width of 80 pixels to generate a background (BG) image. Next, the BG image was subtracted from the RAW image to obtain a background-corrected (COR) image. Noise pixels were removed from the COR image using a median filter (MED, 3×3 kernel, 2 passes). Then the image was thresholded using a cut-off of 20 gray values yielding a binary (BIN) image. The latter was combined with the COR image using a Boolean AND operation^28^ to obtain a masked (MSK) image. In the latter image, the average fluorescence intensity was quantified by dividing the integrated pixel intensity (gray value) by the total number of pixels. This parameter is computationally equivalent to CaIOD/Casum (Table S1) and reflects the average cellular fluorescence intensity of the ROS reporter molecule.

### Data analysis

Image visualization, processing and quantification was carried out using Image Pro Plus^®^ software (Media Cybernetics, Rockville, MD, USA) and custom MATLAB^®^ algorithms (Mathworks, Natick, MA., USA), as described in detail previously^28^. Data visualization and analysis was performed using Origin Pro^®^ (OriginLab, Northampton, MA, USA) and Graphpad PRISM^®^ software (Graphpad Software, San Diego, CA, USA). The number of independent experiments and replicates are indicated by *N* and *n*, respectively. Unless stated otherwise, data from multiple experiments is presented as the mean ± standard error of the mean (SEM) and statistical significance was assessed using an independent two-population Student’s *t*-test with Bonferroni correction (*P < 0.05; **P < 0.01; ***P < 0.001).

## Results

### Galactose specifically induces death of LS fibroblasts in a time-dependent manner

**Fig. 1:**
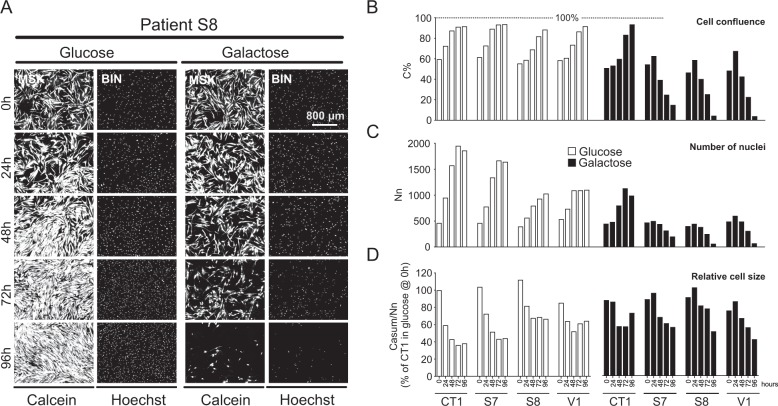
Galactose specifically reduces the viability of LS fibroblasts in a time-dependent manner. **a** Time-dependent effect of glucose and galactose in LS fibroblasts (S8 cells shown as a typical example). The cells were co-stained with Calcein-AM and Hoechst 33258 to visualize the cytosol and nuclei, respectively. Image processing was applied to calculate “masked” (MSK) and black-and-white “binary” (BIN) images (see Results for details). **b** Time-dependent analysis of control (CT1) and LS cells (S7, S8, V1) cultured in glucose-containing or galactose-containing medium (*N* = 1, *n* ≥ 2). The *y*-axis represents C%, the percentage of total pixels in the BIN image that was Calcein-positive. C% is a measure of cell confluence. **c** Same as **b** but now for the number (Nn) of Hoechst-positive objects (nuclei; middle panel), calculated from the Hoechst BIN image. Nn is used as a measure of cell number. **d** Same as **b** but now for the total number of Calcein-positive pixels (Casum; a measure of cell area) divided by the number of nuclei (Nn). This reflects the relative size of the cells (expressed as % of CT1 in glucose @ 0 h). Results for the individual cell lines are presented in Fig. S1, S2, S3, S4

**Fig. 2:**
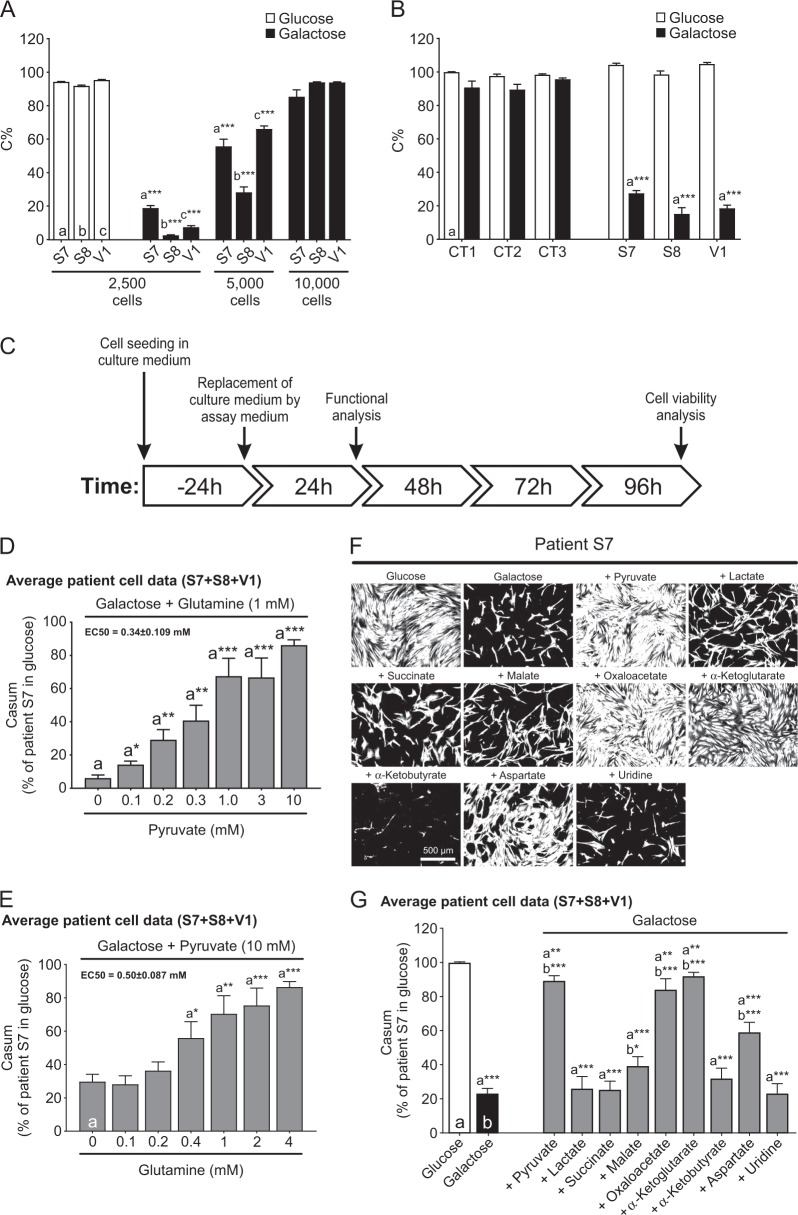
The viability of LS fibroblasts is reduced in galactose medium in a cell density-dependent manner and rescued by specific metabolites. **a** Effect of cell seeding density on the galactose-induced reduction in viability of LS cells (S7, S8, V1; measured at 96 h; *N* = 1, *n* = 8). **b** Specificity of the galactose-induced reduction in viability (2500 cells seeded; measured at 96 h; *N* = 3, *n* ≥ 12) for LS cells relative to CT cells (CT1, CT2, CT3). **c** Experimental protocol used in this study to quantify the effects of galactose and other treatments on control and LS cells. **d** Dose-dependent rescue of the galactose-induced reduction in viability of LS cells (S7, S8, V1) at 96 h by pyruvate in the presence of a constant glutamine concentration (*N* = 3, *n* ≥ 9). The EC50 value was determined by fitting a Hill equation to the data (*R*^2^ = 0.97). **e** Same as **d**, but now for the dose-dependent effect of glutamine in the presence of a constant pyruvate concentration (*N* = 3, *n* ≥ 18). The EC50 value was determined by fitting a Hill equation to the data (*R*^2^ = 0.98). **f** Rescuing effect of specific metabolites on the galactose-induced reduction in LS cell viability (96 h; Calcein MSK images for patient S7 are shown as a typical example): sodium pyruvate (10 mM), sodium l-lactate (10 mM), sodium succinate dibasic hexahydrate (10 mM), l-(-)-malic acid (10 mM), oxaloacetic acid (10 mM), dimethyl α-ketoglutarate (10 mM), 2-ketobutyric acid (10 mM), l-aspartic acid (15 mM), and uridine (1 mM). The “ + ” conditions reflect combined galactose + metabolite treatment. **g** Quantification of the rescuing effect of the metabolites (*N* = 3, *n* ≥ 18) in LS patient cells (S7, S8, V1) at 96 h (*N* = 3, *n* ≥ 18). Statistics: *P < 0.05, *P < 0.01, ***P < 0.001, relative to the marked conditions (**a**, **b**, **c**). Results for the individual cell lines are presented in Fig. S6, S7

Fibroblasts from control subjects (CT1, CT2, CT3) and LS patients with isolated CI deficiency (S7, S8, V1) were routinely cultured in glutamine-containing DMEM without pyruvate to which 5.5 mM glucose or galactose was added. Viability analysis revealed that control cells proliferated in glucose and galactose medium (Fig. 1b), whereas LS cells proliferated in glucose medium but died in galactose medium (Fig. 1a). CT1 and LS cells gradually decreased in size in glucose and galactose medium (Fig. 1d). These results demonstrate that glucose-by-galactose replacement specifically reduces the viability of LS cells relative to CT1 cells in a time-dependent manner. However, performing experiments over a time course of 18 months (Table S2) revealed that S8 patient cells invariably died in galactose medium, whereas death was variably induced in S7 and V1 cells. A correlation was observed between the time point at which a freshly prepared galactose medium was used for the first time and a change in the galactose-induced killing efficiency (Fig. S5). Since a medium change might affect cell growth, we determined whether the LS cell seeding density affected their galactose-induced killing at 96 h. Higher seeding densities protected LS fibroblasts against galactose-induced cell death (Fig. 2a). Therefore, we routinely seeded 2500 cells/well and only analyzed experiments where glucose-by-galactose replacement significantly reduced LS cell viability. This yielded consistent results (Fig. 2b), allowing cell functional and viability analyses (Fig. 2c). Given the similar response to galactose within the control and LS patient cell group (Fig. 2b) we compared CT1 with average LS cell data in the remainder of the study (the full dataset is presented in the Supplement, as indicated in the figure legends).

### Galactose-induced death of LS fibroblasts is rescued by specific metabolites in an oleamide-sensitive manner

**Fig. 3:**
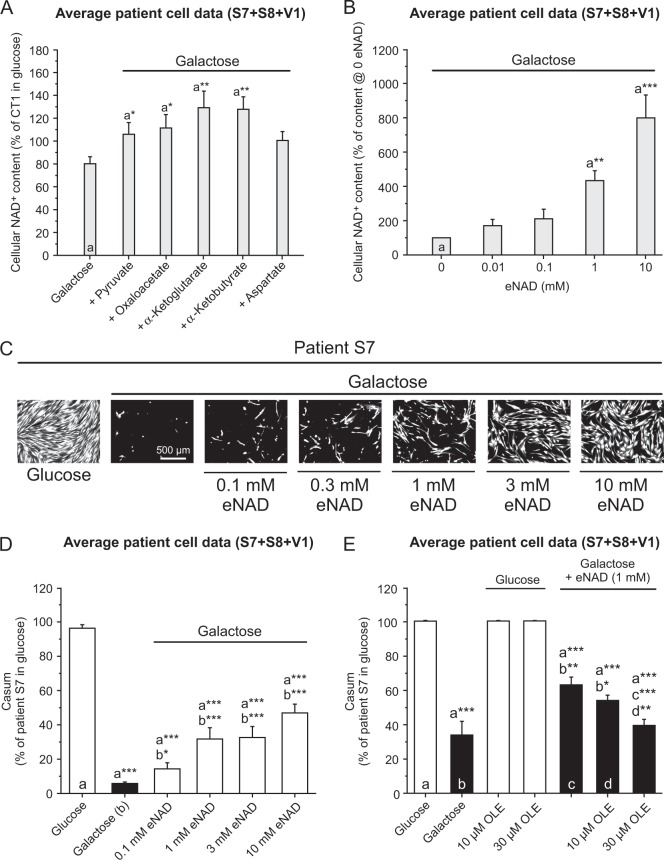
Exogenous NAD^+^ (eNAD) rescues the galactose-induced reduction in cell viability and intracellular NAD^+^ (iNAD) content in LS fibroblasts. **a** Dose-dependent effect of extracellular sodium pyruvate (10 mM), oxaloacetic acid (10 mM), dimethyl α-ketoglutarate (10 mM), 2-ketobutyric acid (10 mM), and l-aspartic acid (15 mM) on intracellular NAD (iNAD) levels in LS cells (24 h; *N* = 1, *n* = 3). **b** Dose-dependent effect of extracellular NAD (eNAD; β-nicotinamide adenine dinucleotide hydrate) on intracellular NAD (iNAD) levels (24 h; *N* = 1, *n* ≥ 6). **c** Dose-dependent rescue of the galactose-induced reduction in viability of LS cells (Calcein MSK images) by eNAD (β-nicotinamide adenine dinucleotide hydrate; S7 shown as a typical example; 96 h). **d** Quantification of the rescuing effect of eNAD in LS patient cells (96 h; *N* = 2 *n* ≥ 9). **e** Dose-dependent inhibition of the rescuing effect of eNAD by oleamide (OLE) in LS patient cells (96 h; *N* = 3, *n* = ≥ 6). Statistics: *P < 0.05, *P < 0.01, ***P < 0.001, relative to the marked condition (**a**, **b**, **c**, **d**). Results for the individual cell lines are presented in Fig. S8

Galactose-induced LS cell death was dose-dependently inhibited by pyruvate in the presence of glutamine and vice versa (Fig. 2d, e). Supplementation of the galactose medium with malate, oxaloacetate, α-ketoglutarate or aspartate also (partially) rescued LS cell death (Fig. 2f, g). Lactate, succinate, α-ketobutyrate or uridine displayed no rescue (Fig. 2f, g). None of the metabolites affected LS cell viability in glucose medium (Fig. S7A). Various rescuing metabolites (e.g., pyruvate, oxaloacetate, α-ketoglutarate) increased cellular NAD^+^ content (iNAD; Fig. 3a). Although α-ketobutyrate also increased iNAD (Fig. 3a), galactose-induced LS cell death was not rescued (Fig. 2g). This demonstrates that the rescuing effects of the above metabolites are not unequivocally linked to increased iNAD levels. Next, we evaluated the effect of extracellular NAD^+^ (eNAD) on iNAD^33^ and cell viability. Adding eNAD to the galactose medium increased iNAD (Fig. 3b) and rescued galactose-induced LS cell death (Fig. 3c, d). This rescue was prevented by oleamide (Fig. 3e), an inhibitor of gap-junction hemichannel-mediated NAD^+^ entry^34^. These findings demonstrate that eNAD application increases iNAD and inhibits galactose-induced death of LS cells via an oleamide-sensitive mechanism.

### Rescue of galactose-induced death of LS fibroblasts by eNAD is enhanced by pyruvate

**Fig. 4:**
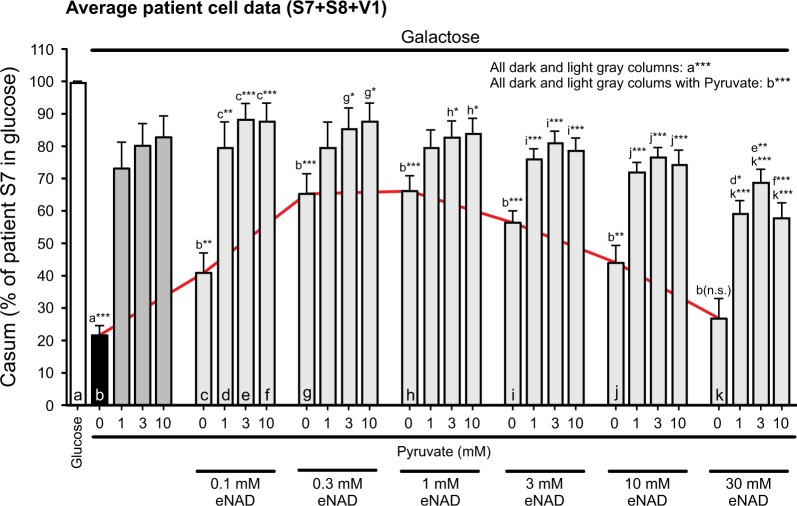
Combined effect of pyruvate and exogenous NAD^+^ (eNAD) on the galactose-induced reduction in cell viability of LS fibroblasts. Effect of pyruvate and eNAD on the viability of LS cells (S7, S8, V1) cultured in galactose medium (*N* = 1, *n* ≥ 9; 96 h). Casum, the number of white pixels in the Calcein BIN image, was expressed as percentage of the total number of pixels for patient S7 in glucose medium. The red line highlights the effect of eNAD in the absence of pyruvate. Statistics: *P < 0.05, *P < 0.01, ***P < 0.001, relative to the marked condition (**a**, **b**, **c**, **d**, **e**, **f**, **g**, **h**, **i**, **j**, **k**). Results for the individual cell lines are presented in Fig. S9

Supplementation with eNAD increased iNAD levels to a greater extent than pyruvate supplementation (Fig. 3a, b). However, pyruvate rescued galactose-induced LS cell death to a greater extent than eNAD (e.g., Figs. 2g, 3d, e). Combination experiments demonstrated that, in the absence of pyruvate, eNAD concentrations up to 1 mM partially rescued galactose-induced LS cell death (Fig. 4; red line). By itself, pyruvate inhibited galactose-induced LS cell death to a greater extent than eNAD (Fig. 4; dark gray bars vs. red line). Moreover, rescue was always larger for pyruvate + eNAD than for eNAD alone (Fig. 4; light gray bars vs. red line). In contrast, pyruvate-induced rescue was not stimulated by the presence of eNAD. These results suggest that the rescue of galactose-induced LS cell death by pyruvate is not primarily due to its iNAD-increasing effect.

### Mitochondrial morphofunctional analysis demonstrates differential effects of pyruvate and eNAD

**Fig. 5:**
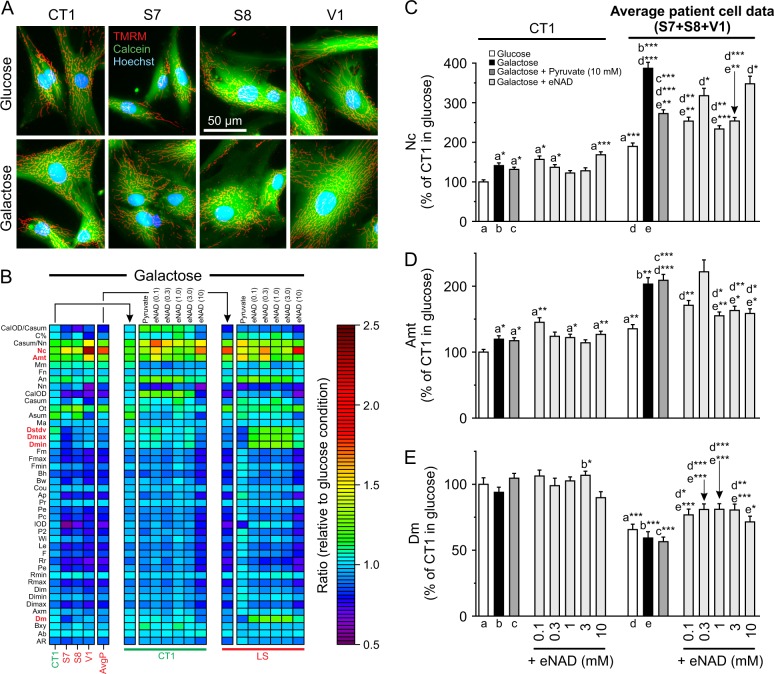
Effect of galactose, pyruvate and exogenous NAD^+^ (eNAD) on mitochondrial morphofunction in LS fibroblasts. CT1 and LS cells (S7, S8, V1) were cultured in glucose or galactose medium for 24 h and analyzed. **a** Typical microscopy images of cells stained with TMRM (mitochondria), Calcein-AM (cytosol) and Hoechst 33258 (nuclei). **b** Heatmap displaying the average value of the morphofunctional descriptors (*y*-axis; normalized on the glucose condition). The left group of panels depict results obtained using the galactose medium (CT1, S7, S8, V1, and average patient data; AvgP).The middle group of panels depict results obtained using the galactose + pyruvate (10 mM) and galactose + eNAD (0.1, 0.3, 1, 3, and 10 mM) medium (CT1 cells). The right group of panels is similar to the middle group of panels but now depicting the average results obtained with LS cells. **c** Values of Nc, representing the the number of mitochondria per cell. **d** Values of Amt, representing the total mitochondrial area per cell. **e** Values of Dm, representing the average mitochondrial TMRM intensity (a measure of the mitochondrial membrane potential Δψ). Statistics: Glucose and galactose conditions (CT1, S7, S8: *N* = 2, *n* = 60; V1: *N* = 1, *n* = 12). Galactose + pyruvate condition (CT1, S7, S8, V1: *N* = 1, *n* = 12). Galactose + eNAD condition (CT1, S7, S8, V1: *N* = 1, *n* = 12; all concentrations). Results for the individual cell lines and detailed data analysis are presented in Fig. S10, S11

High-content microscopy analysis (Fig. 5a) of TMRM/Calcein/Hoechst-stained cells^28^ was applied to analyze mitochondrial morphology and function (“morphofunction”). This yielded 44 descriptor values (Table S1), which were normalized to the glucose condition and visualized using a heatmap (Fig. 5b). Exploratory analysis (Fig. S11) revealed that: (i) mitochondrial morphofunction is differentially affected by galactose in CT1 and LS fibroblasts, and (ii) the galactose-induced phenotypic differences between CT1 and LS cells disappear upon supplementation with eNAD, but not pyruvate. Regression analysis (Fig. S11C-D) was used to highlight the descriptors most differentially altered between CT1 and LS cells (Fig. 5b; red characters). In glucose medium LS cells contained a larger number of mitochondria (Nc) than CT1 cells (Fig. S11C). This difference was enhanced by galactose treatment (Fig. 5c). Pyruvate and eNAD supplementation inhibited the galactose-induced Nc increase in LS cells but not in CT1 cells (Fig. 5c). In glucose medium, the total mitochondrial area per cell (Amt) was higher in LS cells than in CT1 cells and this difference was enhanced by galactose treatment (Fig. 5d). Pyruvate supplementation did not prevent the galactose-induced increase of Amt in LS and CT1 cells, whereas eNAD was effective in LS cells but not in CT1 cells (Fig. 5d). Adding eNAD to the galactose medium increased various descriptors (Dstdv, Dmax, Dmin, and Dm) in LS but not in CT1 cells (Fig. S11E). These parameters are all related to the TMRM fluorescence intensity (Table S1), with Dm representing the average mitochondrial TMRM fluorescence. In glucose medium, LS cells displayed a lower Dm than CT1 cells, indicating a less negative Δψ (Fig. 5e). Galactose treatment in the absence/presence of pyruvate did not affect this difference (Fig. 5e). In LS cells but not CT1 cells, eNAD but not pyruvate supplementation increased Dm, suggesting that eNAD partially restored Δψ (Fig. 5e).

### Pyruvate and eNAD reduce the levels of CM-H_2_DCFDA-oxidizing ROS

**Fig. 6:**
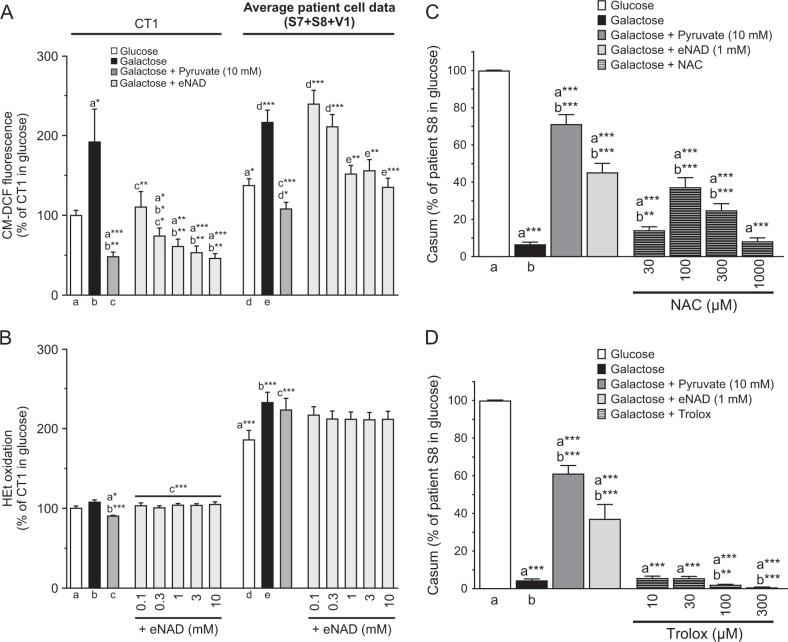
Effect of galactose, pyruvate and exogenous NAD^+^ (eNAD) on cellular ROS levels and of exogenous antioxidants on the viability of LS fibroblasts. CT1 and LS cells (S7, S8, V1) were cultured in glucose or galactose medium and analyzed at 24 h (CM-DCF fluorescence and HEt oxidation) and at 96 h (viability). **a** Effect of the various treatments on cellular ROS levels as reported by CM-DCF fluorescence (*N* = 4, *n* ≥ 36). **b** Same as **a** but now for HEt oxidation (*N* = 4, *n* ≥ 36). **c** Effect of the antioxidant N-acetylcysteine (NAC) on the viability of S8 cells in galactose medium (*N* = 3, *n* = 12). **d** Same as **c** but now for the antioxidant Trolox (*N* = 3, *n* = 12). Statistics: *P < 0.05, **P < 0.01, ***P < 0.001, relative to the marked condition (**a**, **b**, **c**, **d**, **e**). Results for the individual cell lines are presented in Fig. S12

Extracellular pyruvate displays antioxidant properties in human cells^35–38^. In glucose medium, LS cells displayed higher levels of CM-H_2_DCFDA- and HEt-oxidizing ROS than CT1 cells (Fig. 6a, b). Galactose treatment increased CM-H_2_DCFDA oxidation to a similar level in CT1 and LS cells (Fig. 6a) without affecting HEt oxidation (Fig. 6b). Similarly, pyruvate and eNAD lowered CM-H_2_DCFDA oxidation (Fig. 6a) without affecting HEt oxidation (Fig. 6b). In S8 cells the glutathione precursor N-acetylcysteine (NAC) prevented galactose-induced LS cell death, whereas the vitamin E-derivative Trolox (6-hydroxy-2,5,7,8-tetramethylchroman-2-carboxylic acid) was ineffective (Fig. 6c, d). These results suggest that galactose-induced cell death co-involves CM-H_2_DCFDA-oxidizing and NAC-sensitive ROS.

### Cellular ATP content decreases in galactose medium and is normalized by eNAD but not by pyruvate in LS cells

**Fig. 7:**
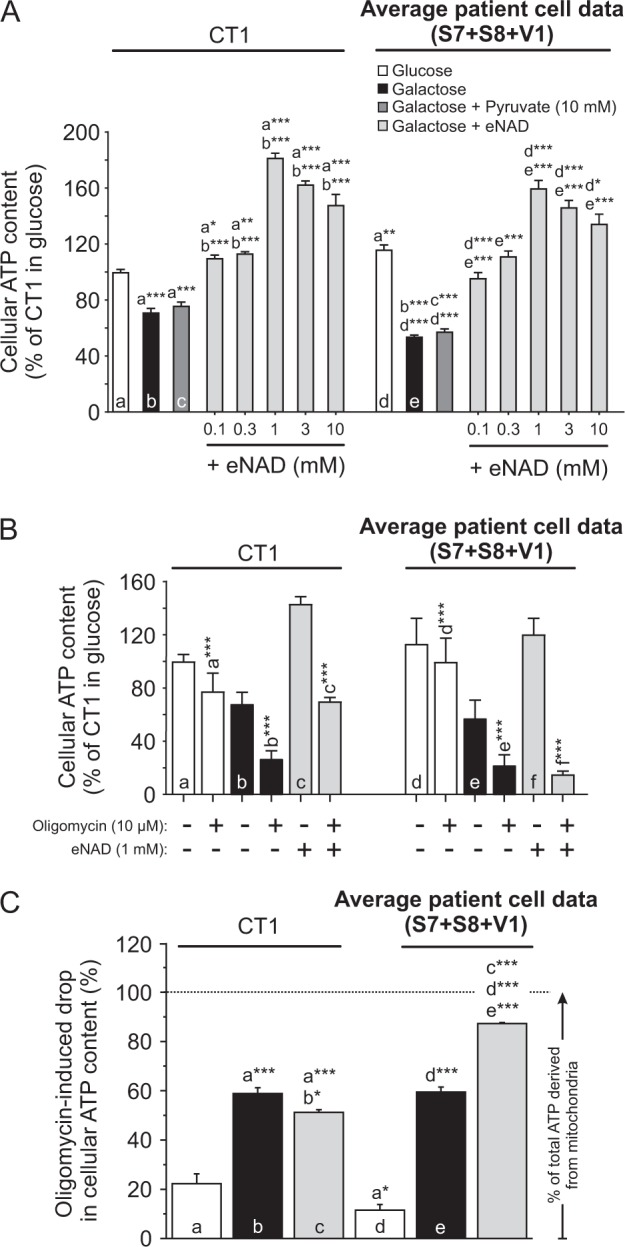
Effect of galactose, pyruvate and exogenous NAD+ (eNAD) on cellular ATP content and mitochondrial ATP production. CT1 and LS cells (S7, S8, V1) were cultured in glucose- or galactose medium for 24 h and analyzed. **a** Effect of the various treatments on the average cellular ATP content in control (CT1) and LS cells (*N* = 5, *n* ≥ 45). **b** Effect of the various treatments (see legend in **a**), in the absence and presence of Oligomycin (10 µM, 30 min), on the average cellular ATP content in control (CT1) and LS cells (*N* = 4, *n* ≥ 36). **c** Effect of the various treatments (see legend in **a**) on the Oligomycin-induced drop in cellular ATP content, reflecting the contribution of OXPHOS-derived ATP production (calculated from the data in **b**). Statistics: *P < 0.05, **P < 0.01, ***P < 0.001, relative to the marked condition (**a**, **b**, **c**, **d**, **e**, **f**). Results for the individual cell lines are presented in Fig. S13

Pyruvate and eNAD differentially affected Δψ in galactose-cultured LS cells (Fig. 5e), potentially impacting on mitochondrial ATP production. In glucose medium, the ATP content of LS fibroblasts exceeded that of CT1 cells (Fig. 7a). Glucose-by-galactose replacement reduced ATP content to a greater extent in LS cells than in CT1 cells (Fig. 7a). In both CT1 and LS cells, eNAD supplementation normalized the galactose-induced reduction in ATP content, whereas pyruvate did not (Fig. 7a). Preventing mitochondrial ATP production by the F_o_F_1_-ATPase (CV) inhibitor Oligomycin A (OLI) reduced the ATP content of CT1 and LS cells under all conditions (Fig. 7b). This demonstrates that mitochondrial ATP production is active. In glucose medium, quantification of this ATP reduction revealed that mitochondria contribute twofold less to total ATP levels in LS cells than in CT1 cells (Fig. 7c). Given their higher ATP level (Fig. 7a; white bars), this suggests that glycolysis is more active in LS cells. Galactose treatment induced a threefold and sixfold increase in the mitochondrial contribution to total ATP content in CT1 and LS cells, respectively (Fig. 7c). Addition of eNAD did not affect the mitochondrial ATP contribution in CT1 but further increased this parameter in LS cells (Fig. 7c).

## Discussion

**Fig. 8:**
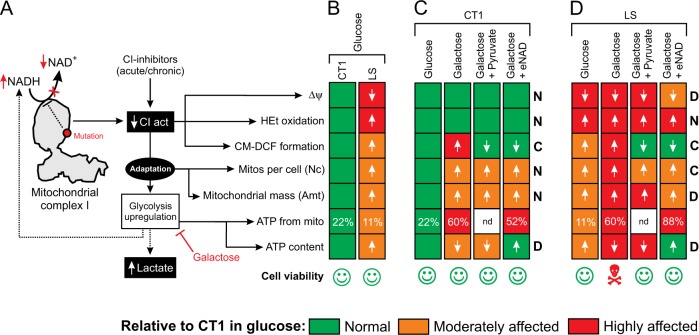
Differential effects of galactose, pyruvate and eNAD on live-cell functional parameters in CT1 and LS fibroblasts. **a** Mutations in mitochondrial complex I (CI) or chemical CI inhibition reduce the conversion of NADH into NAD^+^ and CI catalytic activity (CIact), associated with increased NADH and reduced NAD^+^ levels (“reductive stress”). The reduced CI activity is associated with alterations in mitochondrial membrane potential (Δψ), the levels of HEt- and H_2_DCFDA-oxidizing ROS, the number of mitochondria per cell (Nc), mitochondrial mass (Amt), mitochondrial ATP production and cellular ATP levels. As an adaptation to CI deficiency, glycolysis is upregulated leading to increased production of NADH and lactate. In this study, glucose in the culture medium was replaced by galactose to inhibit glycolysis upregulation. **b** Functional changes associated with glucose-by-galactose replacement in control (CT1) fibroblasts. The magnitude of each change is color coded in green (no effect), orange (moderately affected) or red (highly affected). Cell viability is indicated by pictograms. **c** Same as **b** but now also including the effects of pyruvate and exogenous NAD^+^ (eNAD) supplementation on CT1 cells. For each measured parameter, it is indicated whether both supplements displayed a common effect (“C”), differential effect (“D”) or no effect (“N”). The glucose condition was reproduced from **b**. **d** Same as **c**, but now for fibroblasts from Leigh Syndrome (LS) patients with isolated CI deficiency. The glucose condition was reproduced from **b**

Activation of glycolysis during CI dysfunction can mask deficiency-induced aberrations in cell physiology (Fig. 8a). Here we prevented this activation by culturing CT and LS fibroblasts in pyruvate-free media containing galactose. In glucose medium, LS fibroblasts exhibited a partially depolarized Δψ, increased HEt and H_2_DCFDA oxidation and altered mitochondrial morphology (Fig. 8b), compatible with our previous findings^39^. In addition, we here demonstrate that LS cells display a higher ATP content than CT1 fibroblasts and derive less of their total ATP from mitochondria. This suggests a mechanism in which: (i) the CI-deficient state stimulates glycolytic lactate and ATP generation, thereby reducing the mitochondrial contribution to cellular ATP content, and (ii) NADH levels increase as the combined result of glycolytic upregulation and hampered mitochondrial NADH-to-NAD^+^ conversion by CI (Fig. 8a).

### Galactose induces specific death of LS cells

Glucose-by-galactose replacement induced the specific death of LS fibroblasts without affecting CT1 cell viability, providing that a sufficiently low cell seeding density was used. We speculate that cell-cell adhesion might play a role in counteracting the galactose effect^40^ and/or death-suppressing pathways might become activated at high cell densities^41^. Galactose treatment invariably reduced the viability of S8 fibroblasts with a low residual CI activity (18% of lowest control value) whereas variable results were obtained with S7 and V1 cells displaying a moderate reduction in CI activity (68–64%). This is compatible with previous findings^26^ and suggests a mechanism in which the degree of CI deficiency determines the extent of compensatory glycolysis activation and, as a consequence, the cellular sensitivity to glucose-by-galactose replacement. Indeed, CI inhibition dose-dependent increased lactate production in mouse fibroblasts^42^ and NADH-cytochrome *c* reductase activity inversely correlated with the L/P ratio in primary fibroblasts from OXPHOS-deficiency patients (linear correlation: *R* = −0.61; *p* = 0.01; data from ref. ^19^.

### Pyruvate rescues galactose-induced death of LS cells

Galactose only induced specific death of LS fibroblasts in the absence of pyruvate. Since pyruvate is normally produced by the glycolysis pathway^43^, this suggests that: (i) the reduced glycolytic flux in galactose-treated cells induces pyruvate shortage, and (ii) this pyruvate shortage is responsible for the specific galactose-induced death of LS cells. Pyruvate-mediated rescue of galactose-induced LS cell death was glutamine-dependent. However, although glutamine was present in all galactose media it did not prevent LS cell death in the absence of pyruvate. In HeLa cells, glutamine fuels OXPHOS-mediated mitochondrial ATP production via the TCA cycle, providing more than half of the ATP in the presence of glucose and virtually all ATP upon replacing glucose by galactose^44^. In addition, inhibition of the mitochondrial pyruvate carrier in cancer cells activated glutamate dehydrogenase and rerouted the glutamine metabolism to generate oxaloacetate and acetyl-CoA, thereby sustaining TCA cycle function^45^. This situation might also be present in our galactose-treated LS cells, where the glycolytic pyruvate production flux is expected to be low. Alternatively, in case of OXPHOS-deficient cells with mitochondrial DNA (mtDNA) mutations, it was proposed that impaired NADH utilization by the mitochondrial ETC triggers reductive carbonylation of glutamine in the cytosol catalyzed by malate dehydrogenase 1 ^46^. This study proposed a mechanism in which reduced mitochondrial NADH turnover inhibits the mitochondrial malate-aspartate shuttle (MAS), leading to cytosolic NADH accumulation. The latter then induces cytosolic reductive carbonylation of glutamine, which provides carbons for NADH-coupled MDH1 and thereby regulates NAD^+^ redox state and enhances the activity of the glycolysis enzyme GAPDH. This then increases glycolytic flux to enhance ATP production in the cytosol^46^. However, since this mechanism requires a highly active glycolysis pathway it is unlikely that it explains the glutamine-dependence of the pyruvate rescue of galactose-induced LS cell death observed in our experiments. Previous evidence suggests that inhibited cell proliferation during ETC disruption is rescued by pyruvate supplementation via restoration of NAD^+^/NADH balance mediated by lactate dehydrogenase in the cytosol^9,47^. Compatible with this mechanism, we observed that pyruvate slightly increased cellular NAD^+^ content. However, pyruvate also displays antioxidant activity. Here, pyruvate rescue of galactose-induced LS cell death was paralleled by normalization of the galactose-induced increase in CM-H_2_DCFDA-oxidizing ROS levels (Fig. 8d). In contrast, the increased levels of HEt-oxidizing ROS in LS cells were neither stimulated further by galactose treatment nor affected by pyruvate. This suggests that pyruvate might rescue galactose-induced LS cell death by lowering the levels of CM-H_2_DCFDA-oxidizing ROS. Supporting this idea, pyruvate protected human fibroblasts against H_2_O_2_-induced cell death, by lowering CM-H_2_DCFDA-oxidizing ROS levels and preventing Δψ depolarization^48^. Related to this, three other molecules that rescued galactose-induced LS cell death in the current study (pyruvate, oxaloacetate, and α-ketoglutarate) also reduced the levels of CM-H_2_DCFDA-oxidizing ROS and protected against hydrogen peroxide (H_2_O_2_)-induced toxicity^37^. Similarly, non-rescuing molecules in the current study (lactate, succinate, malate, and α-ketobutyrate) were also ineffective in the H_2_O_2_-induced toxicity model^37^. This suggests that (part of) the rescuing effects of pyruvate, oxaloacetate, and α-ketoglutarate is due to their antioxidant properties. Although glutamine also can act as an (in)direct antioxidant^49^, its presence in the galactose medium did not prevent LS cell death. This means that it displays no antioxidant properties in our experimental system and/or its medium concentration is too low. Functionally, pyruvate supplementation did not affect Δψ, Nc, Amt or the decreased ATP content in galactose-treated CT1 cells (Fig. 8c). In galactose-treated LS cells, pyruvate slightly reduced Nc but did not restore Δψ, Amt or cellular ATP content (Fig. 8c). Therefore we propose that pyruvate does not rescue galactose-induced LS cell death by restoring mitochondrial function but by its ability to prevent the galactose-induced increase CM-H_2_DCFDA-oxidizing ROS levels. This means that, under galactose conditions, pyruvate rescue of LS viability requires TCA fueling by glutamine to sustain biomolecule synthesis and cell proliferation^44,45^.

### eNAD rescues galactose-induced death of LS cells

For the metabolites tested in this study, their ability to rescue the galactose-induced death of LS cells was not unequivocally paralleled by an iNAD increase. As mentioned above, this likely relates to fact that several of these metabolites also can act as antioxidants (pyruvate, oxaloacetate, and α-ketoglutarate). However, supplementation of the galactose medium with eNAD increased iNAD^33^ and dose-dependently rescued LS cells from galactose-induced death. Oleamide dose dependently and completely inhibited the eNAD-induced rescue of galactose-induced death in LS cells, suggests that eNAD exerts its protective effects by increasing iNAD via gap-junction hemichannels. Combined treatment with pyruvate and eNAD revealed that pyruvate alone rescued galactose-induced LS cell death to a larger extent than eNAD alone. In addition, rescue was always greater for combined pyruvate and eNAD supplementation than for eNAD alone. Remarkably, eNAD increased iNAD levels to a much greater extent than pyruvate. These results suggest that the rescuing effect of pyruvate is not due to its iNAD-increasing effect, which supports the above conclusion that pyruvate prevents galactose-induced LS cell death by acting as an antioxidant. Similar to pyruvate, eNAD lowered the galactose-induced increase in CM-H_2_DCFDA-oxidizing ROS levels in galactose-treated CT1 (Fig. 8c) and LS cells (Fig. 8d). Although this suggests that iNAD directly lowers these ROS levels, the latter was not observed in HeLa cells^33^. At the functional level, and similar to pyruvate, eNAD supplementation did not affect the normal Δψ, the normal HEt oxidation, the moderately increased number of mitochondria per cell, the moderately increased mitochondrial mass or the moderately decreased ATP content in galactose-treated CT1 cells (Fig. 8c). In contrast, eNAD fully restored the galactose-induced reduction in ATP content of CT1 cells. In the case of galactose-treated LS cells, and in sharp contrast to pyruvate, eNAD partially restored the depolarized Δψ and fully restored the galactose-induced reduction in ATP content (Fig. 8d).

## Conclusions

Our results establish a cell-based strategy for intervention testing and analysis of the pathophysiological consequences and adaptive responses in CI deficiency. We propose a mechanism in which pyruvate rescues the galactose-induced death of LS cells by directly lowering the levels of CM-H_2_DCFDA-oxidizing and NAC-sensitive ROS, during which glutamine (and not pyruvate) is used for TCA cycle fueling. In the case of eNAD, we conclude that it rescues galactose-induced death of LS cells by partially restoring Δψ depolarization and fully normalizing their greatly reduced cellular ATP content. This is compatible with CI-deficient LS cells suffering from galactose-induced reductive stress. Whether NAD^+^ lowers the levels of CM-H_2_DCFDA-oxidizing ROS by reducing mitochondrial ROS production or via (indirectly) acting as an antioxidant remains to be determined.

## Electronic supplementary material

Supplement
