# Supplementary material for: Rescue from galactose-induced death of Leigh Syndrome patient cells by pyruvate and NAD+
Source: Cell Death Dis. 2018 Nov 14;9(11):1135. doi: 10.1038/s41419-018-1179-4 (PMC6235972; doi:10.1038/s41419-018-1179-4)
Supplement: Supplementary file 1 — Supplement [file 41419_2018_1179_MOESM1_ESM.pdf]

A. SUPPLEMENTARY TABLES

Table S1: Meaning of descriptors extracted from Calcein/Hoechst/TMRM images

Nr[#]	Reporter	Descriptor	Symbol	Definition/meaning of descriptor[&]
1	TMRM	**Aspect ratio**	AR	Ratio between major axis and minor axis of an ellipse equivalent to object: measure of mitochondrial length.
2	TMRM	**Area/Box**	Ab	The ratio between the area of an object and the area of its bounding box.
3	TMRM	**Box XonY**	Bxy	Ratio between width and height of object's bounding box.
4	TMRM	**Density mean**	Dm	Average intensity of object (grayvalue): average mitochondrial intensity of TMRM signal.
5	TMRM	**Axis minor**	Axm	Length of minor axis of ellipse with same moments of order 1 and 2 as object (pixels).
6	TMRM	**Diameter max**	Dimax	Length of longest line joining two points of object's outline and passing through the object's centroid (pixels).
7	TMRM	**Diameter min**	Dimin	Length of shortest line joining two points of object's outline and passing through the object's centroid (pixels).
8	TMRM	**Diameter mean**	Dim	Average length of diameters measured at 2 degree intervals and passing through the object's centroid.
9	TMRM	**Radius max**	Rmax	Minimum distance between object's centroid and outline (pixels)
10	TMRM	**Radius min**	Rmin	Maximum distance between object's centroid and outline (pixels)
11	TMRM	**Perimeter ellipse**	P1	The perimeter of the ellipse surrounding the outline of each object (pixels)
12	TMRM	**Radius ratio**	Rr	Ratio between Rmax and Rmin
13	TMRM	**Roundness**	F	Perimeter2/(4*π*Area; a.k.a. Formfactor or F): measure of mitochondrial length and degree of branching.
14	TMRM	**Length**	Le	Feret diameter (caliper length) along the major axis of the object (pixels).
15	TMRM	**Width**	Wi	Feret diameter (caliper length) along the minor axis of the object (pixels).
16	TMRM	**Perimeter2**	P2	Chain code length of the outline (pixels)
17	TMRM	**IOD**	IOD	Integrated optical density of all objects (grayvalue): sum of all mitochondrial TMRM intensity values.
18	TMRM	**Perimeter convex**	Pc	Perimeter of the convex outline of the object (pixels)
19	TMRM	**Perimeter**	P3	Length of the object's outline (pixels)
20	TMRM	**Perimeter ratio**	Pr	Ratio of convex perimeter to perimeter
21	TMRM	**Area polygon**	Ap	Area included in the polygon defining the objects outline (same polygon as used for Perimeter)
22	TMRM	**Count**	Cou	Size weighted object "count" (number of objects).
23	TMRM	**Box width**	Bw	Width of the object's bounding box (pixels).
24	TMRM	**Box height**	Bh	Height of the object's bounding box (pixels).
25	TMRM	**Feret min**	Fmin	Smallest feret (caliper) length (pixels).
26	TMRM	**Feret max**	Fmax	Longest feret (caliper) length (pixels).
27	TMRM	**Feret mean**	Fm	Average feret (caliper) length (pixels).
28	TMRM	**Density min**	Dmin	Minimum density inside object (grayvalue).
29	TMRM	**Density max**	Dmax	Maximum density inside object (grayvalue).
30	TMRM	**Density std dev**	Dstdv	Standard deviation of intensity or density inside object (grayvalue).
31	TMRM	**Margination**	Ma	The distribution of intensity between the center of an object and the edge of the object.
32	TMRM	**Area sum**	Asum	Total area of the mitochondrial objects (pixels). The size of individual mitochondrial objects (Am) is given by Asum/Ot.
33	TMRM	**Objects total**	Ot	Total number of objects: total number of mitochondria.
34	Calcein	**Area sum**	Casum	Total area of the objects (pixels).
35	Calcein	**IOD sum**	CaIOD	Integrated pixel intensity of the objects (grayvalue)
36	Hoechst	***Nn***	Nn	Total number of objects: total number of nuclei/cells (assuming 1 nuclear object per cell).
37	Hoechst	**H. area**	An	Average area of the objects (pixels): average area of the nuclei.
38	Hoechst	**H. roundness**	Fn	Roundness of objects: describing nuclear shape.
39	Derived	**Cell mito ratio**	Mm	Given by: Asum/Casum, a measure of mitochondrial mass.
40	Derived	**Norm mito area**	Amt	Given by: Asum/Nn; equalling the total mitochondrial area (in pixels) per cell.
41	Derived	**Norm mito number**	Nc	Given by: Ot/Nn; equalling the average number of mitochondrial objects per cell. Asum/Ot equals the area (size) of individual mitochondrial objects (Am).
42	Derived	**Norm cyto area**	Casum/Nn	Average size of object (pixels): a measure of average cell size.
43	Derived	**Confluence (%)**	C%	Percentage image pixels that are cellular calculated from Casum; a measure of cell confluence.
44	Derived	**Calcein fluoresence**	CaIOD/Casum	Average intensity (grayvalues) per cellular pixel.

[#]Adapted from: **Blanchet et al., Sci. Rep., 2015; Iannetti et al., Nat. Protoc., 2016.** [&]As defined in the Image Pro Plus software. If the measurement unit is not specified in the definition column, it is Arbitrary Units (A.U.).

Table S2: Analysis of experimental variability in glucose and galactose viability experiments

	DATE			MEDIUM				PN				Casum in glucose (% of patient S7 in glucose)				Casum in galactose (% of patient S7 in glucose)			
**#**	**Y**	**D.M.**	**P**	**DP**	**A**	**SB**	**O**	**CT1**	**S7**	**S8**	**V1**	**CT1**	**S7**	**S8**	**V1**	**CT1**	**S7**	**S8**	**V1**
1	1	14.03	1	10.03	4	#1	A	16	16	15	14	98.7	100.0	97.5	99.5	99.0	100.3	28.7	6.7
2	1	14.03	2	10.03	4	#1	A	16	16	15	14	97.4	97.9	93.3	96.8	96.6	98.4	25.6	3.2
3	1	18.03		14.03	4	#1	A	17	17	16	15	93.2	100.0	95.3	98.4	95.3	49.8	8.6	74.1
4	1	29.03		25.03	4	#1	A	19	19	18	17	98.4	100.0	95.3	100.4	98.1	31.9	8.9	7.8
5	1	16.04	1	29.03	19	#1	A	17	17	18	15	97.1	99.9	91.8	100.1	89.4	31.4	4.5	16.6
6	1	16.04	2	29.03	19	#1	A	17	17	18	15	98.4	99.9	94.8	100.7	77.2	21.8	4.5	35.7
7	1	18.04	1	29.03	21	#1	A	18	18	19	16	96.9	100.0	94.2	99.7	97.0	19.4	6.6	94.9
8	1	18.04	2	29.03	21	#1	A	18	18	19	16	96.7	99.9	88.7	98.9	96.5	15.4	3.9	84.6
9	1	21.04		29.03	24	#1	A	19	19	20	17	95.6	100.3	97.5	98.9	96.6	12.3	13.1	61.1
10	1	24.05	1	19.05	5	#1	A	17	18	19	17	94.6	98.3	99.7	101.5	89.8	21.6	32.9	16.0
11	1	24.05	2	19.05	5	#1	A	17	18	19	17	116.3	109.1	117.2	119.1	67.0	13.6	30.7	6.4
12	1	24.05	3	19.05	5	#1	A	17	18	19	17	152.7	99.9	153.0	155.9	30.3	25.3	26.4	15.6
13	1	07.06		03.06	4	#1	A	--	22	21	19	--	100.0	99.4	99.6	--	76.1	24.1	38.6
14	1	14.06		09.06	5	#1	A	--	17	16	16	--	100.0	102.1	103.4	--	10.3	2.7	9.9
15	1	28.06		24.06	4	#1	B	18	16	16		98.6	100.0	82.2	--	98.0	41.8	1.5	--
16	1	02.07		02.07	0	#1	B	14	15	14	18	95.5	100.0	94.2	92.1	94.0	47.2	2.8	5.7
17	1	19.09		15.09	4	#2	B	14	15	14	16	95.1	100.0	90.4	90.3	94.3	21.1	2.2	3.1
18	1	26.09		15.09	11	#2	B	15	16	17	15	97.6	100.0	98.9	98.7	97.5	17.7	6.1	8.6
19	1	01.10		15.09	16	#2	B	--	17	16	18	--	100.0	101.2	100.6	--	38.3	13.0	19.0
20	1	10.10	1	15.09	25	#2	B	--	18	19	17	--	100.0	97.2	94.6	--	14.5	3.8	4.7
21	1	10.10	2	15.09	25	#2	B	--	18	19	17	--	100.0	96.3	94.1	--	18.9	4.2	3.6
22	1	15.10	1	15.09	30	#2	B	18	19	17	18	95.5	100.0	98.9	99.5	97.2	7.2	1.9	1.4
23	1	15.10	2	15.09	30	#2	B	18	19	17	18	96.8	100.0	98.7	99.4	95.7	5.7	2.5	2.8
24	1	18.10		15.09	33	#2	B	--	18	19	20	97.8	100.1	95.1	95.2	93.0	6.7	3.2	4.2
25	2	07.03		16.02	21	#2	C	--	12	12	15	--	100.0	100.3	89.6	--	15.0	9.2	3.1
26	2	14.03	1	10.03	4	#2	C	12	13	12	16	98.6	100.0	86.0	97.4	97.2	31.4	2.5	7.7
27	2	14.03	2	10.03	4	#2	C		13	12	16	--	100.0	70.4	99.6	--	32.1	3.2	14.4
28	2	15.04	1	11.04	4	#2	C	14	17	16	16	98.8	100.0	107.6	73.7	92.7	16.3	4.9	28.8
29	2	15.04	2	11.04	4	#2	C	--	17	16	16	--	100.0	122.4	110.2	--	28.9	5.3	35.3
30	2	13.05		11.04	35	#2	C	13	16	15	13	99.6	100.0	83.6	88.2	87.8	14.4	0.4	2.2
31	2	16.05		12.05	4	#2	C	--	17	15	13	--	100.0	96.3	100.7	--	38.0	9.7	22.9
32	2	21.08	1	03.08	18	#2	A	--	17	18	19	--	100.0	99.7	99.8	--	59.0	5.9	78.4
33	2	21.08	2	03.08	18	#2	A	--	17	18	19	--	100.0	100.4	100.2	--	44.9	2.2	61.2
34	2	29.08	1	03.08	26	#2	A	--	16	14	15	--	100.0	98.5	100.1	--	64.0	8.8	85.3
35	2	29.08	2	03.08	26	#2	A	--	16	14	15	--	100.0	97.6	100.7	--	57.7	7.3	82.5
36	2	04.09	1	31.08	5	#3	A	--	17	15	16	--	100.1	99.2	100.0	--	99.5	5.0	91.5
37	2	04.09	2	31.08	5	#3	A	--	17	15	16	--	103.9	100.4	100.0	--	101.8	3.7	96.3
**Total number of wells:**												**105**	**171**	**171**	**163**	**139**	**211**	**211**	**199**
**Total number of days (independent experiments):**												**16**	**25**	**25**	**24**	**16**	**25**	**25**	**24**

Abbreviations: -- = not measured; A = Age; D.M = Day.Month; DP = Date prepared; O = Operator; P = Plate; PN = Passage Number; SB = Serum Batch; Y = Year.

B. SUPPLEMENTARY FIGURES

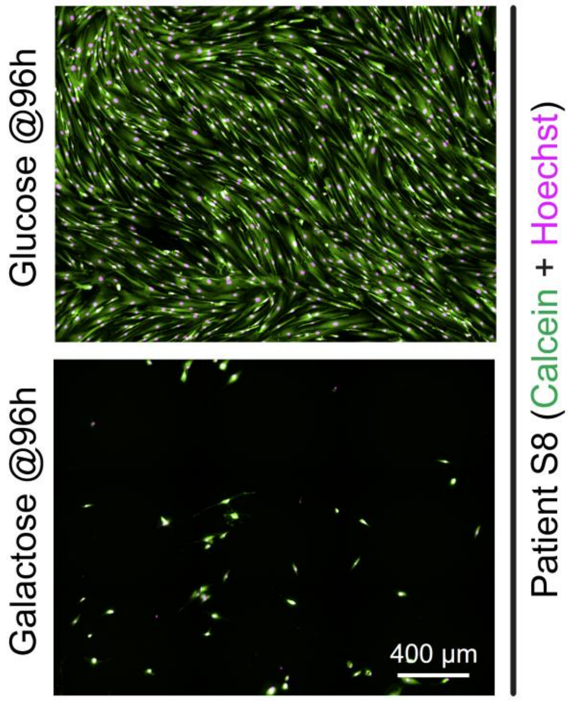

Supplementary figure S1: Magnification of the MSK image in panel A (96 h) of figure 1A for S8 patient fibroblasts. It depicts the co-localization of Calcein (green) and Hoechst (purple) staining for the glucose and galactose condition.

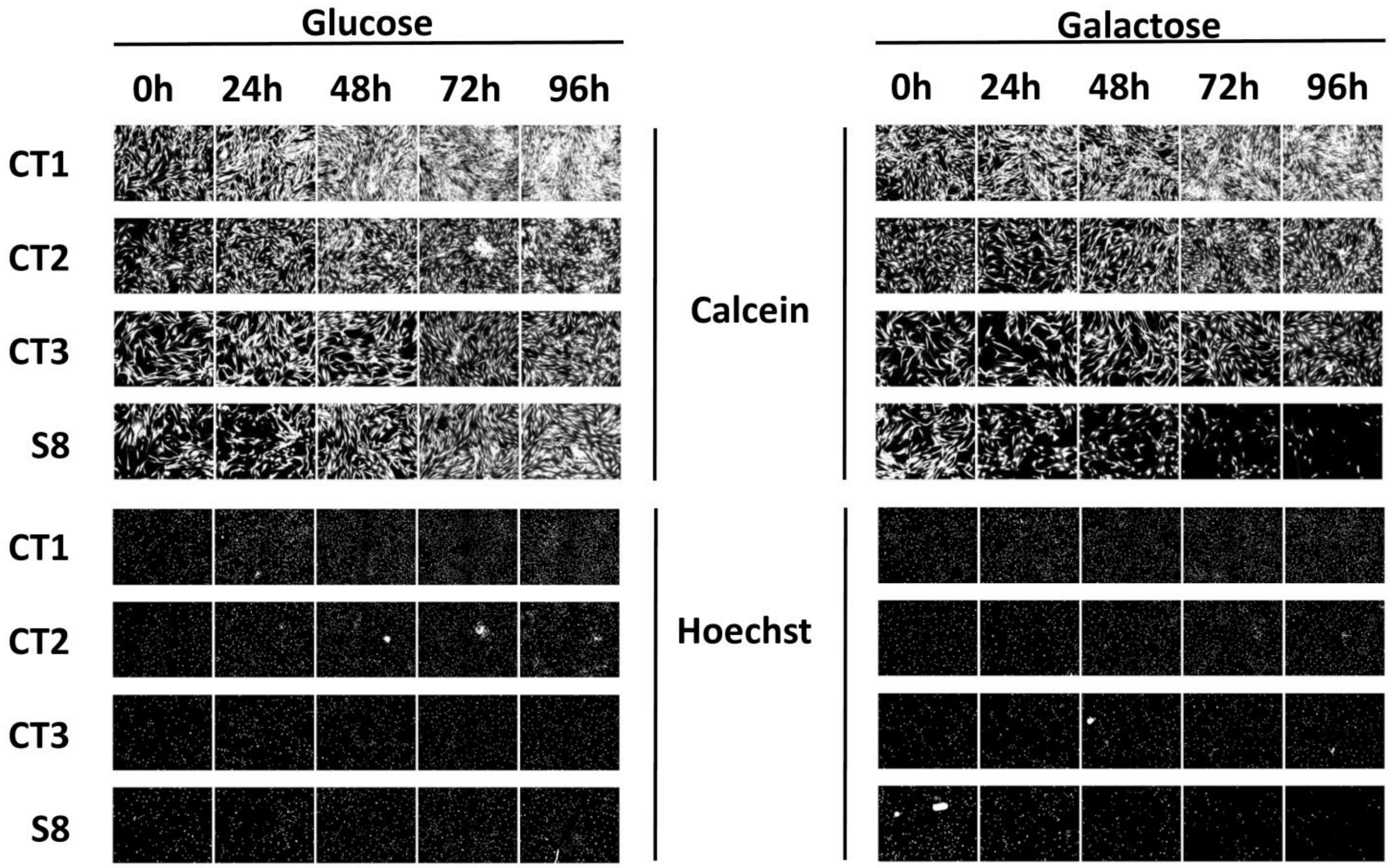

Supplementary figure S2: Galactose specifically reduces the viability of LS fibroblasts in a time-dependent manner. The cells were co-stained with Calcein-AM and Hoechst 33258 to visualize the cytosol and nuclei, respectively. Image processing was applied to calculate "masked" (MSK) and black-and-white "binary" (BIN) images (see Results for details). A time-dependent analysis was performed (N=3, n≥2) of control (CT1, CT2, CT3) and LS cells (S8) cultured in glucose-medium (**left panel**) or galactose-medium (**right panel**). The viability of LS fibroblasts was specifically reduced in the galactose medium in a time-dependent manner, whereas this phenomenon was not observed in the glucose medium. A quantitative analysis is presented in **Supplementary figure S3**.

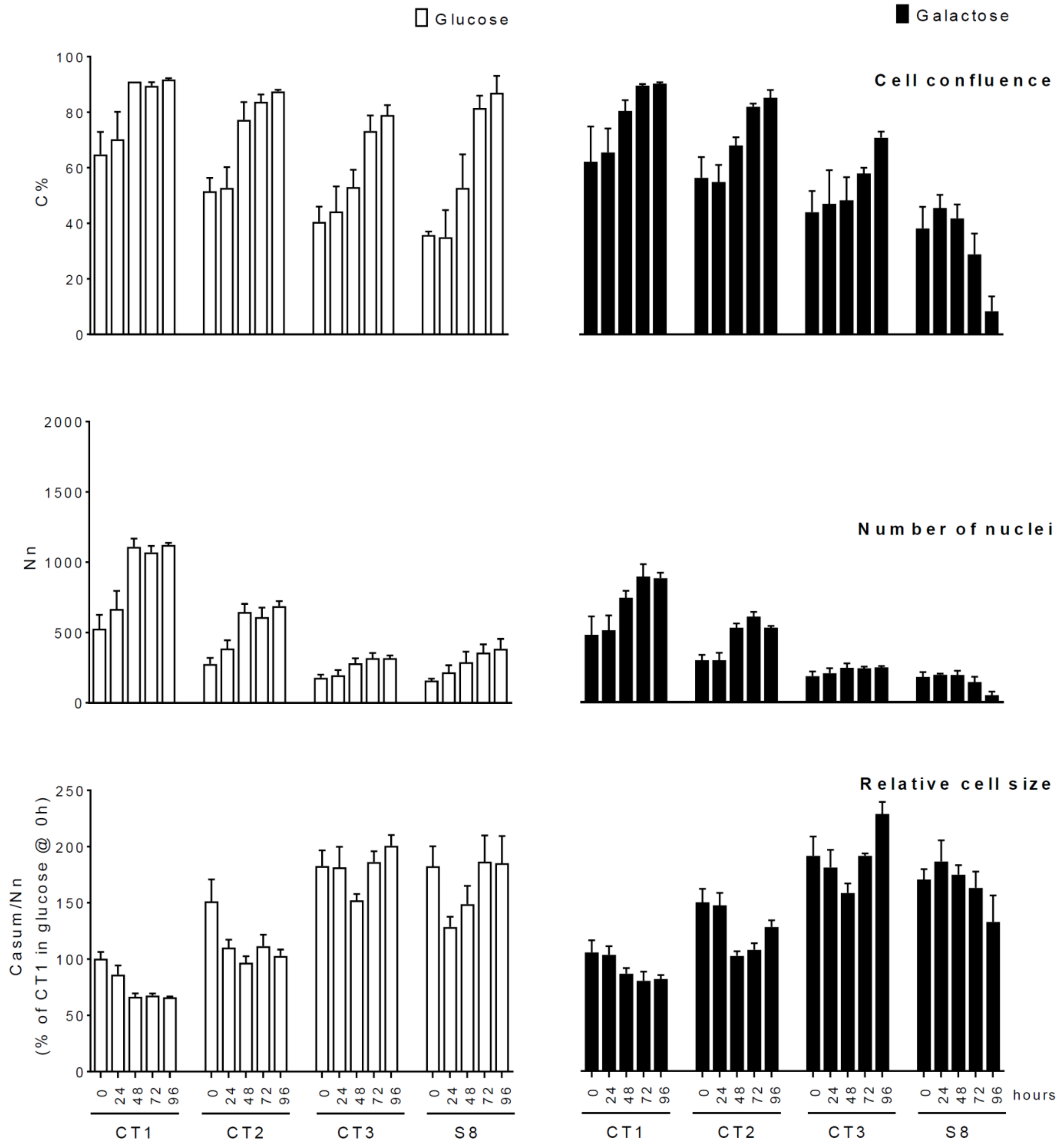

Supplementary figure S3: Galactose specifically reduces the viability of LS fibroblasts in a time-dependent manner. Quantification of the experiments described in **Supplementary figure S2**. **Upper panel:** the y-axis represents C%, the percentage of total pixels in the BIN image that was Calcein-positive. C% is a measure of cell confluency. **Middle panel:** The y-axis represents the number (Nn) of Hoechst-positive objects (nuclei), calculated from the Hoechst BIN image. Nn is used as a measure of cell number. **Lower panel**: The y-axis reflects the total number of Calcein-positive pixels (Casum; a measure of cell area) divided by the number of nuclei (Nn). This reflects the relative size of the cells (expressed as % of CT1 in glucose @ 0 h). See Results and **figure 1** for details.

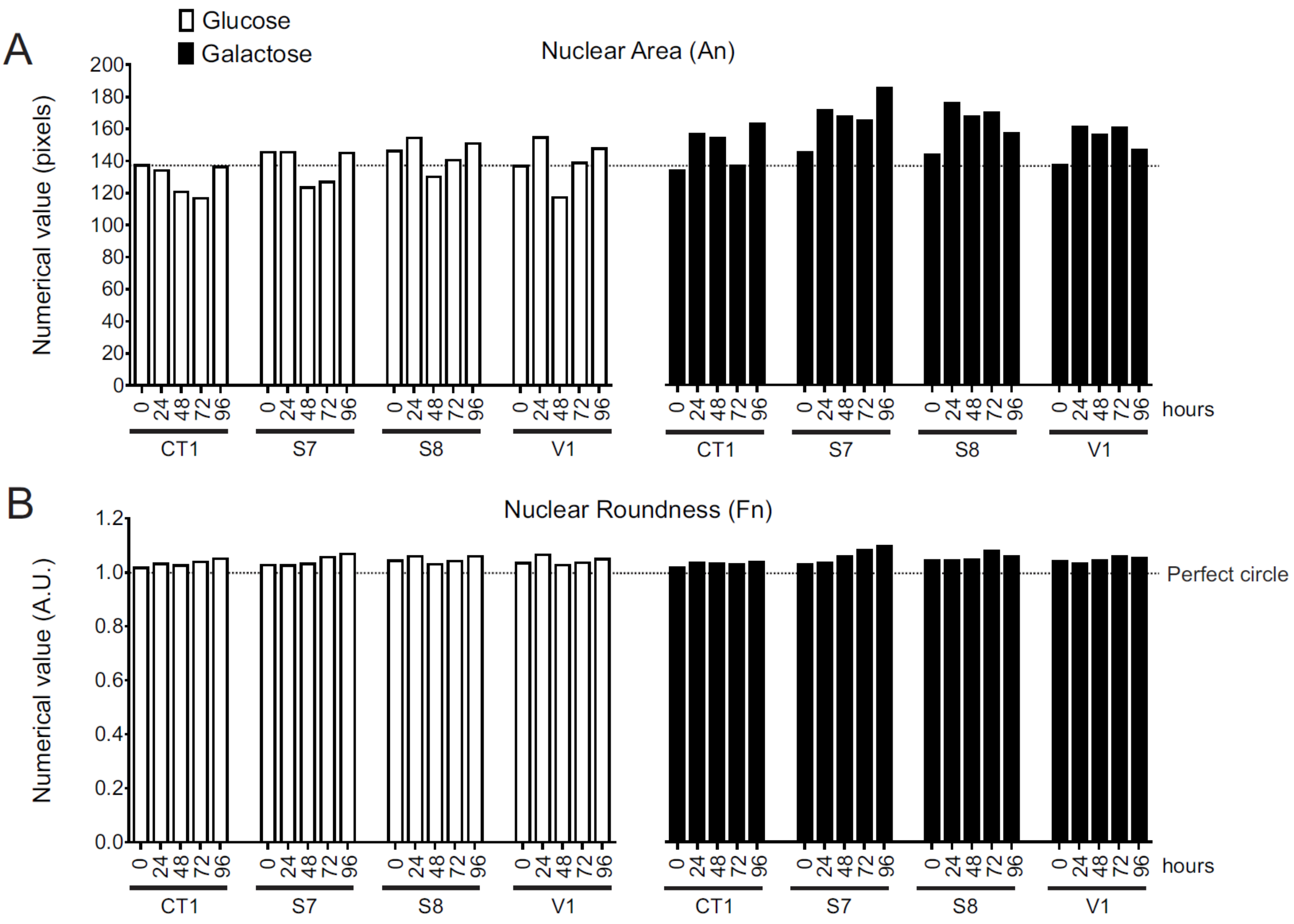

Supplementary figure S4: Nuclear morphological parameters derived from the Hoechst binary images in figure 1. (**A**) Time dependent effect of glucose and galactose in CT1 and LS patient cells (S7, S8, V1) on the Nuclear Area (An) given in pixels (N=1, n≥2). (**B**) Same as panel A but now for the Nuclear Roundness (or Formfactor; Fn). Dotted lines indicate the value of An in CT1 cells at 0 hours (panel A) and Fn (*i.e.* a value of 1) for a perfect circle in panel B (see also **Table S1**). An was larger in galactose than in glucose medium for both CT1 and LS cells, whereas Fn was not affected. This demonstrates that nuclear fragmentation is absent for all cell lines in glucose and galactose medium and, therefore, Nn is a true measure of the number of nuclei.

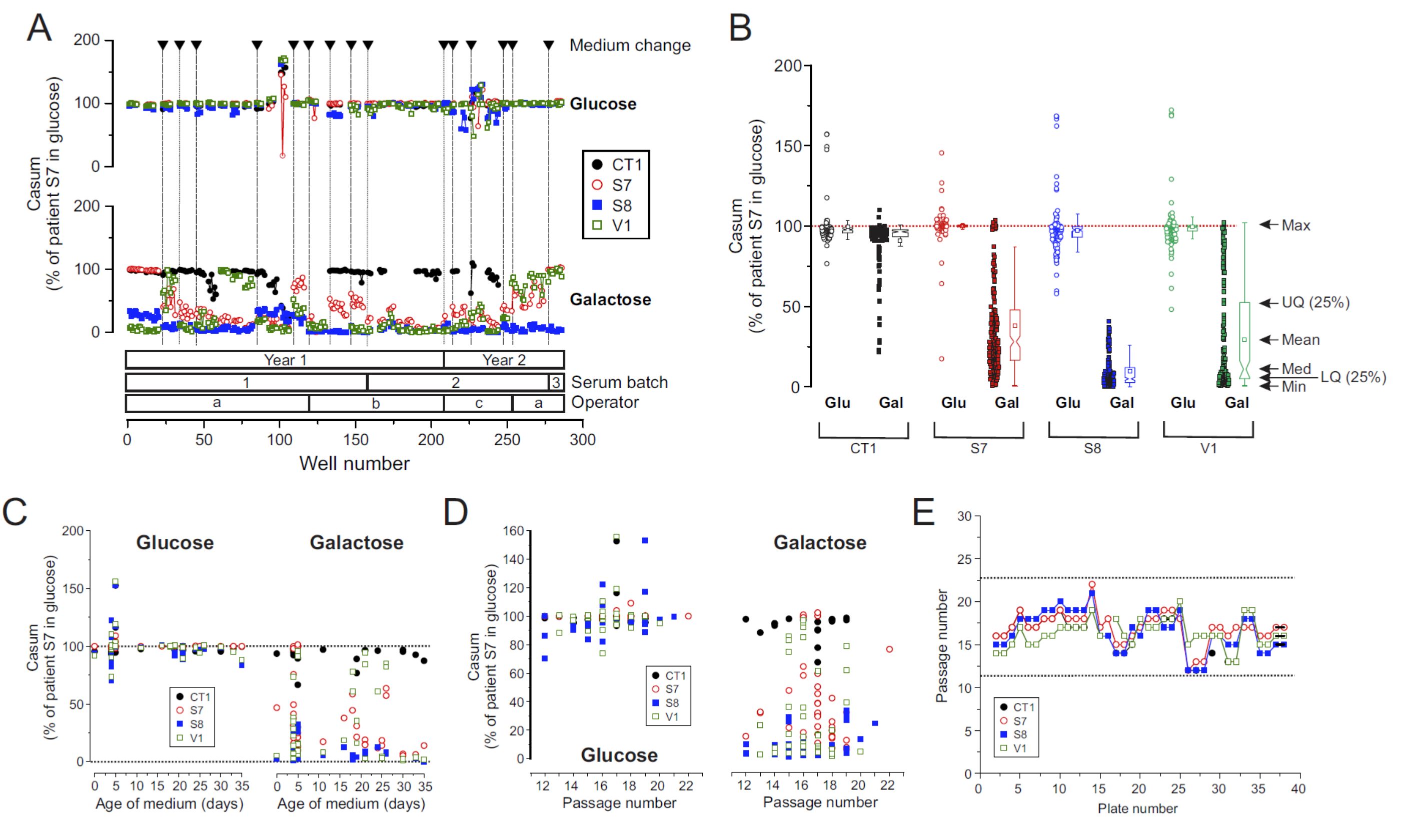

Supplementary figure S5: Effect of galactose treatment on the viability of CT1 and LS cells in multiple experiments performed over a time period of 18 months. The numerical data for this figure is provided in **Table S2**. (**A**) The effect of galactose treatment (96 h) on cell viability was quantified in multiple experiments performed using different batches of serum ("serum batch") by different researchers ("Operator") over a period of 18 months ("year 1" and "year 2"). Black triangles and dotted vertical lines indicate the time points when a freshly prepared galactose medium was used for the first time. The upper and lower halves of the graph displays the effect of culturing in glucose and galactose medium, respectively. A total number of 105-199 wells (individual symbols; x-axis) were analyzed in 16-25 independent experiments (plates) for control (CT1; black circles), NDUFS7 ("S7", red circles), NDUFS8 ("S8", blue squares) and NDUFV1 ("V1", green squares) fibroblasts. Since S7 cells were present in every experiment, Casum was normalized on the value obtained for S7 cells in glucose, since this condition was present on every plate. (**B**) Alternative representation of the galactose data depicted in panel A using a box and whisker plot. The latter indicates the highest value (Max), upper 25% quartile (UQ), average value (Mean), median value (Med), lower 25% quartile (LQ) and lowest value (Min) in the data set. (**C**) Cell viability (96 h) of the various cell lines cultured in glucose (left panel) and galactose medium (right panel) as a function of the age of the culture medium. (**D**) Similar to panel C, but now for cell passage number. (**E**) Passage number of the various cell lines used in the experiments.

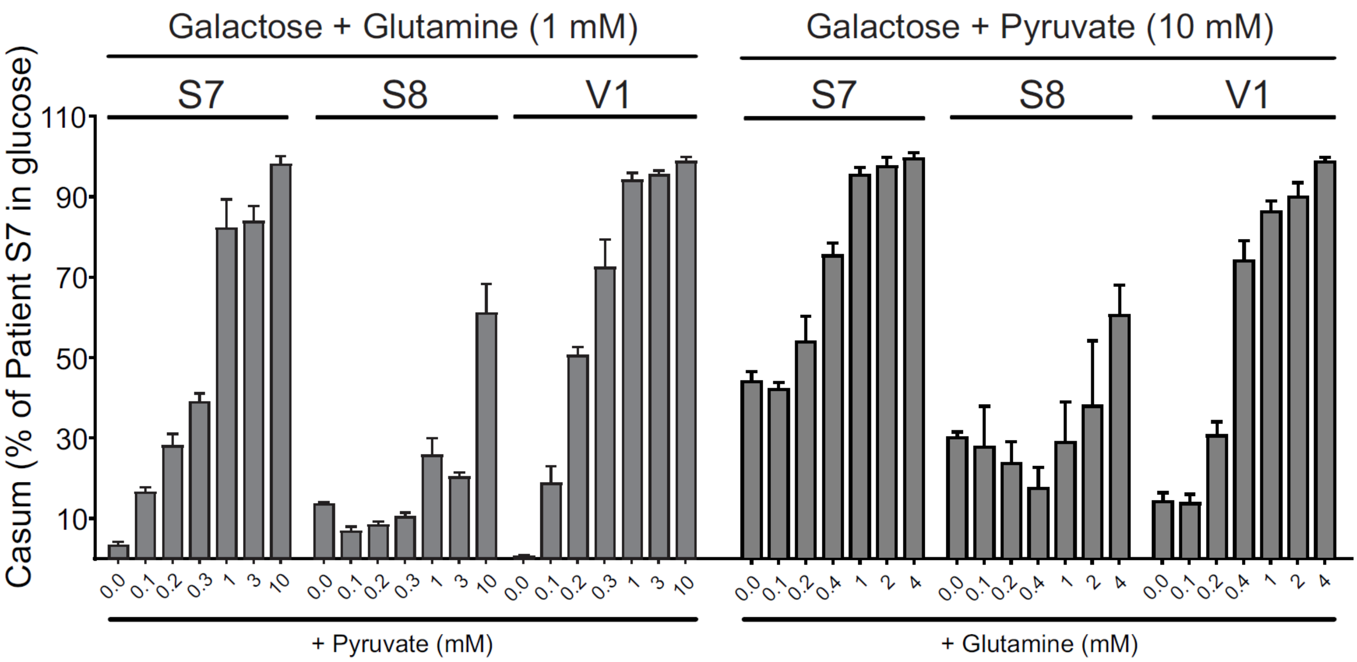

Supplementary figure S6: Data for individual cell lines (S7, S8, V1) used to create figure 2D and E. See Results for details.

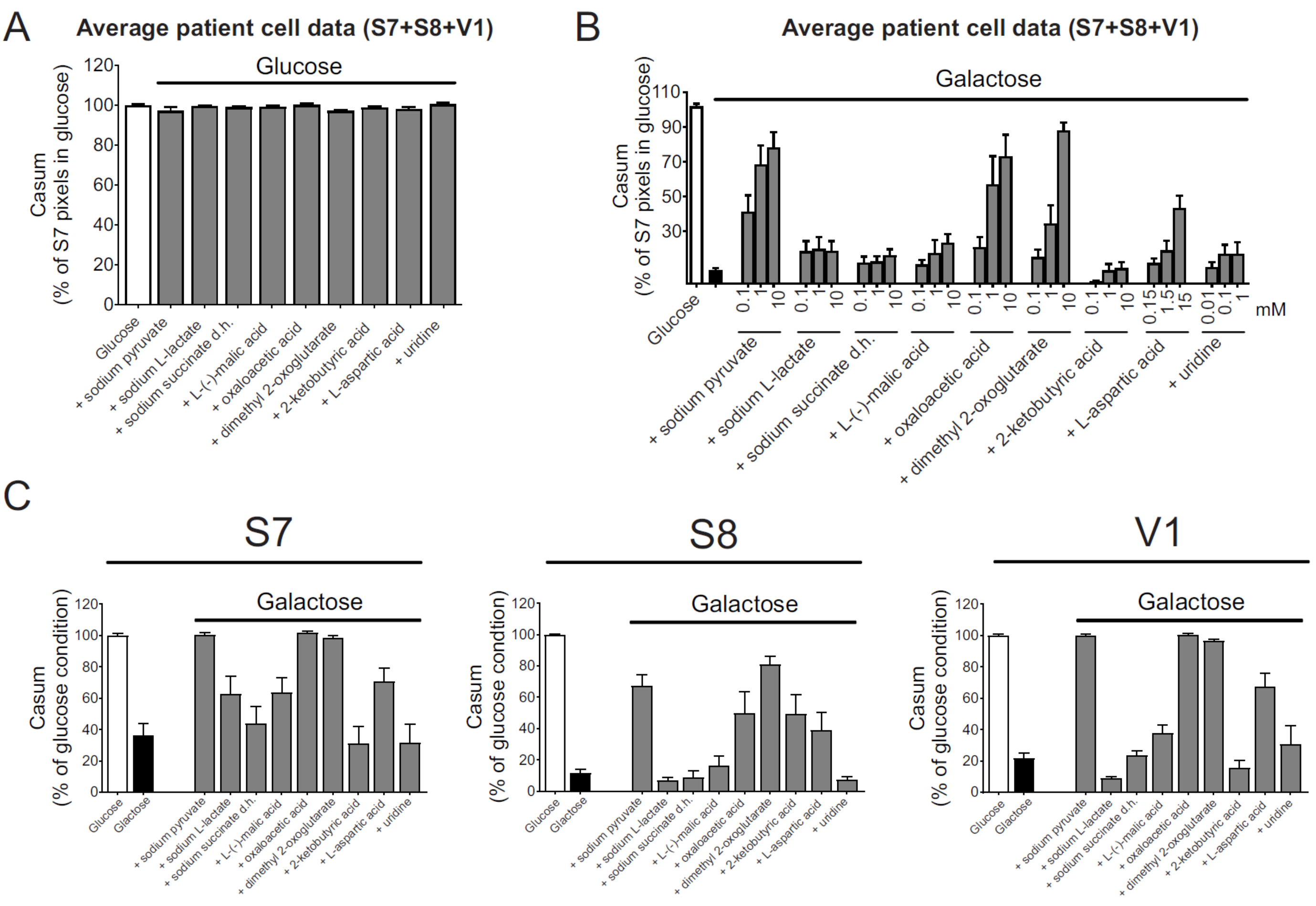

Supplementary figure S7: Data for individual cell lines (S7, S8, V1) used to create figure 2G. See Results for details.

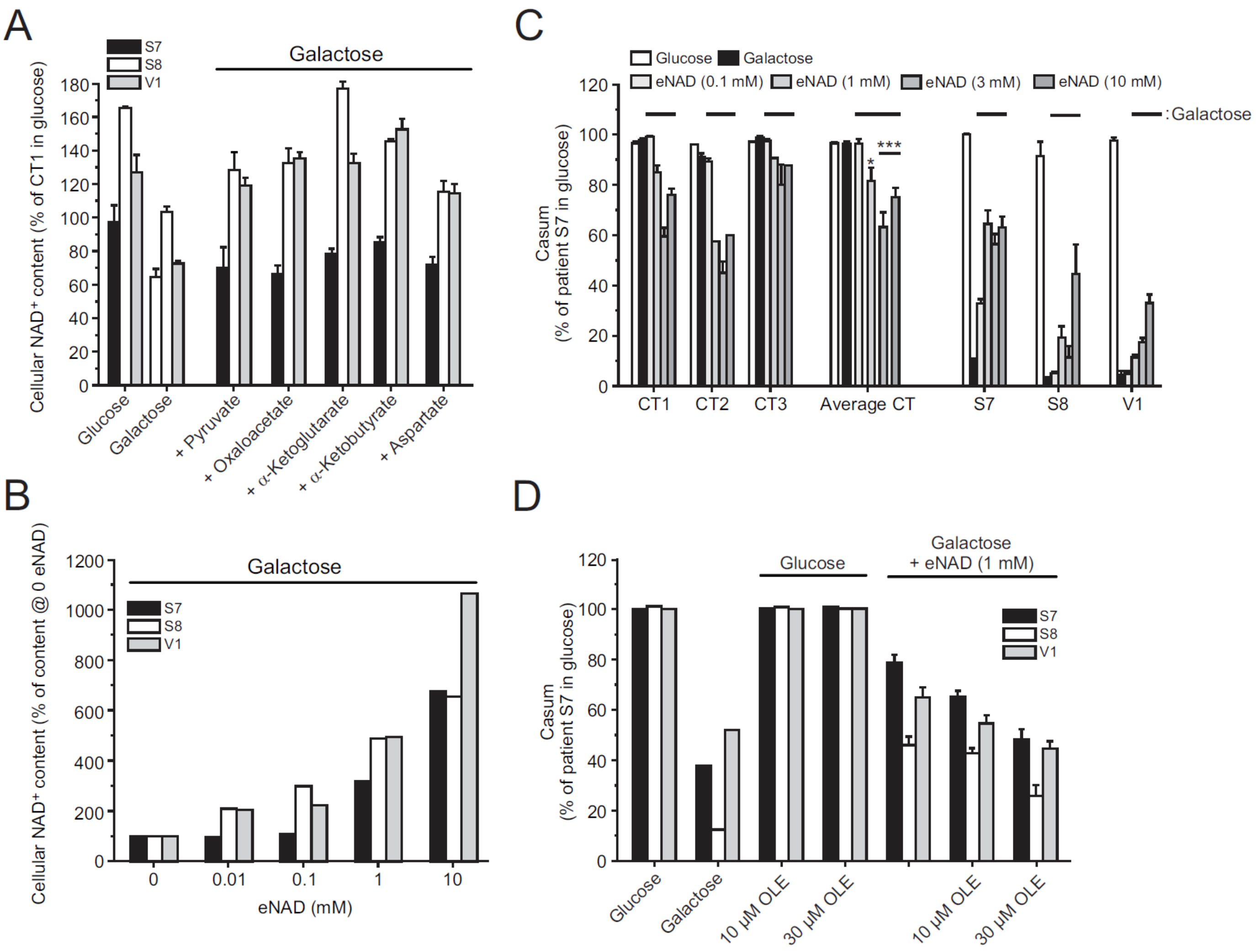

Supplementary figure S8: Data for individual cell lines (S7, S8, V1) used to create figure 3A, B, D and E. See Results for details.

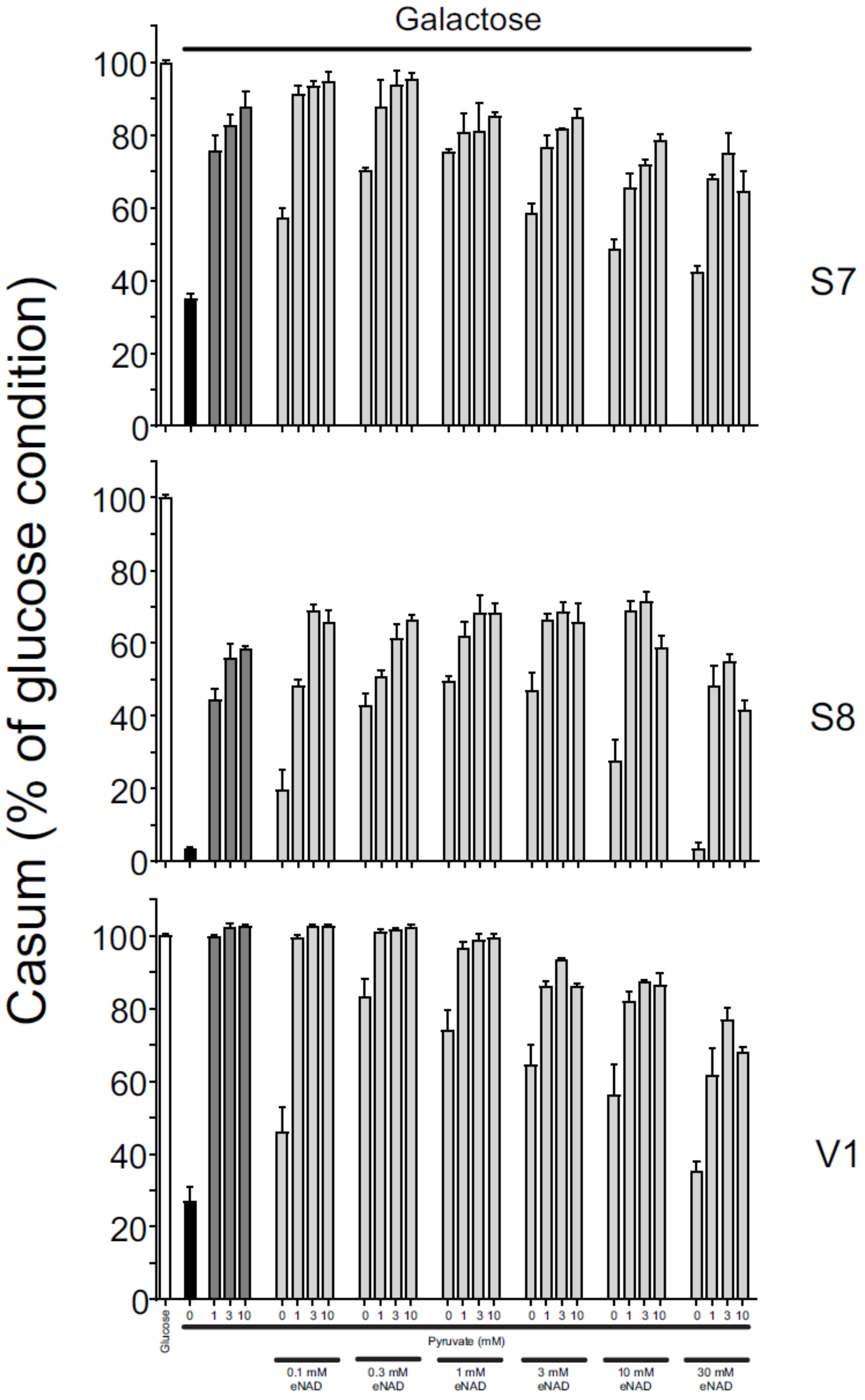

Supplementary figure S9: Data for individual cell lines (S7, S8, V1) used to create figure 4. See Results for details.

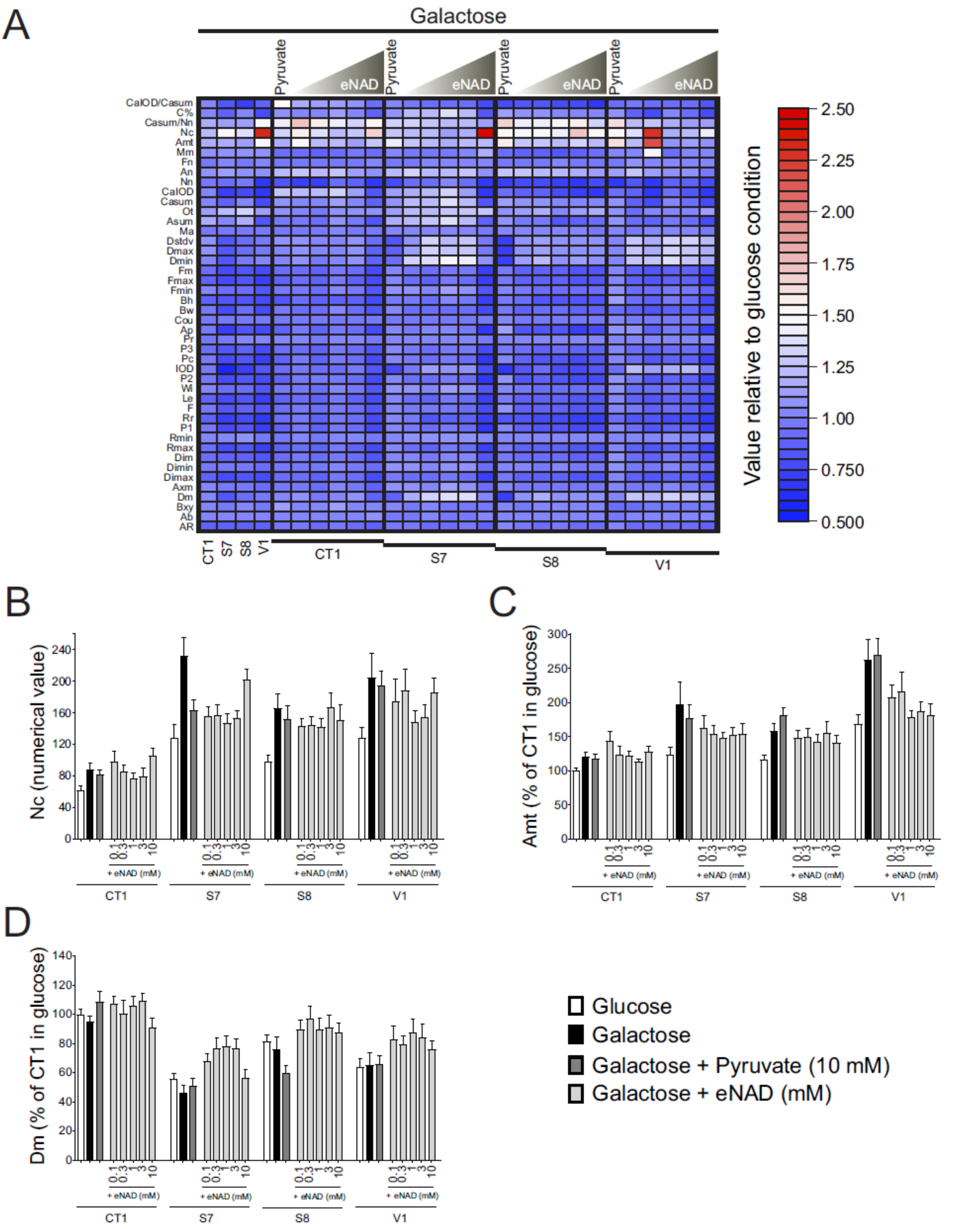

Supplementary figure S10: Data for individual cell lines (CT1, S7, S8, V1) used to create figure 5B, C, D and E. See Results for details.

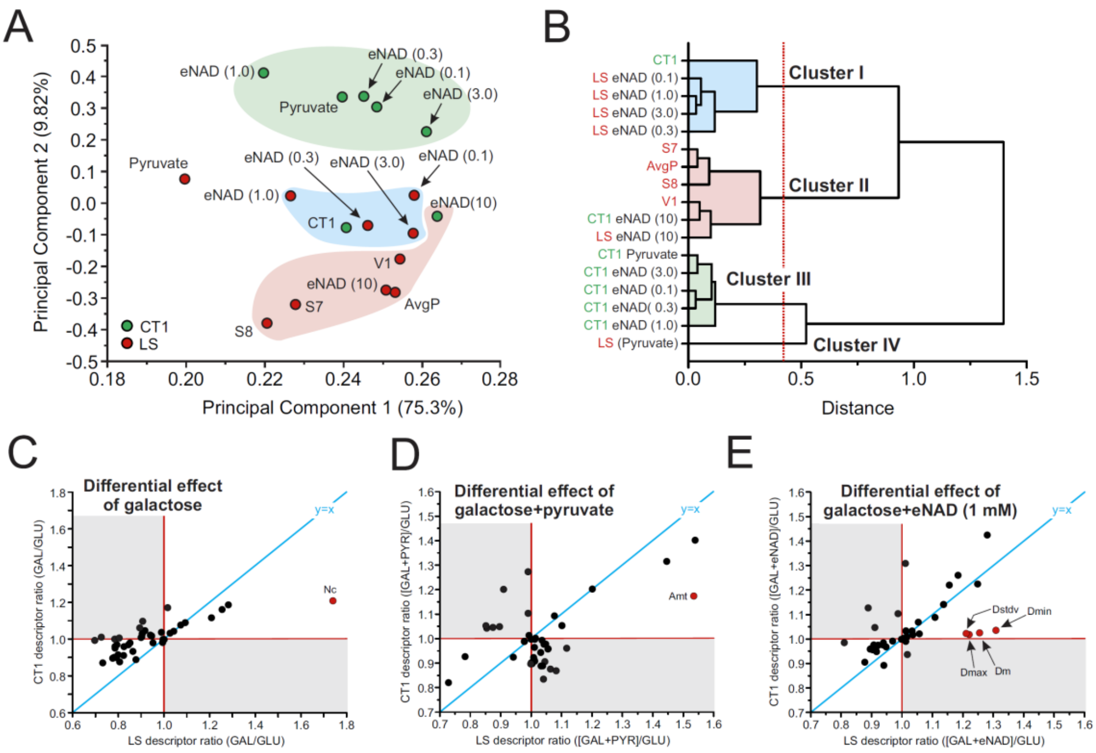

Supplementary figure S11: Analysis of the mitochondrial morpofunctional data in figure 5B. (**A**) Principal component analysis (PCA) of the data depicted in **figure 5B**. The colour of the symbols indicates CT1 (green) and LS patient (red) data. Three groups (marked by different colors) were detected in the data using cluster analysis (see panel B). (**B**) Cluster analysis of the data depicted in **figure 5B** (using the Ward clustering method and correlation as a distance measure). (**C**) To gain more detailed insight into the morphofunctional consequences of the various treatments, we determined which descriptors were differentially affected the most in CT1 *vs*. LS cells. To this end the average numerical value for each descriptor for CT1 cells was plotted as a function of its numerical value in LS cells. Descriptors for CT1 that are not affected by the treatment fall on the horizontal red line. Descriptors for LS cells that are not affected by the treatment fall on the vertical red line. Descriptors that are similarly affected by the treatment in CT1 and LS cells fall on the line y=x (blue). Grey boxes mark the regions were the treatments induced opposite changes in CT1 and LS cells. In case of the differential effect of galactose (relative to the glucose condition) on CT1 (y-axis) and LS patient cells (x-axis), this analysis yielded Nc (the number of mitochondria per cell) as the most extreme differential descriptor (red symbol). (**D**) Same as panel C, but now for the differential effect of galactose + pyruvate on LS and CT1 cells. The most extreme differential descriptor was Amt (red symbol). (**E**) Same as panel C, but now for the differential effects of galactose + 1 mM eNAD on LS and CT1 cells. The most extreme differential descriptors were Dstdv, Dmax, Dm and Dmin (red symbols). **Panel A and B** demonstrate that galactose treatment induces a different phenotype in CT1 and LS cells, as exemplified by the CT1 condition being part of cluster I (blue), whereas the S7, S8 and V1 conditions are part of cluster II (pink). Moreover, CT1 and eNAD-treated LS cells (0.1, 0.3, 1.0 and 3.0 mM) part of cluster I, demonstrating that their mitochondrial morphofunctional phenotypes are highly similar. In contrast, LS cells cultured in galactose medium supplemented with a higher eNAD concentration (10 mM) or pyruvate were not part of cluster I. This means that the galactose-induced phenotypic difference between CT1 and LS cells disappears upon supplementation with eNAD (up to a concentration of 3.0 mM), whereas a high eNAD concentration (10 mM) and pyruvate do not have this effect.

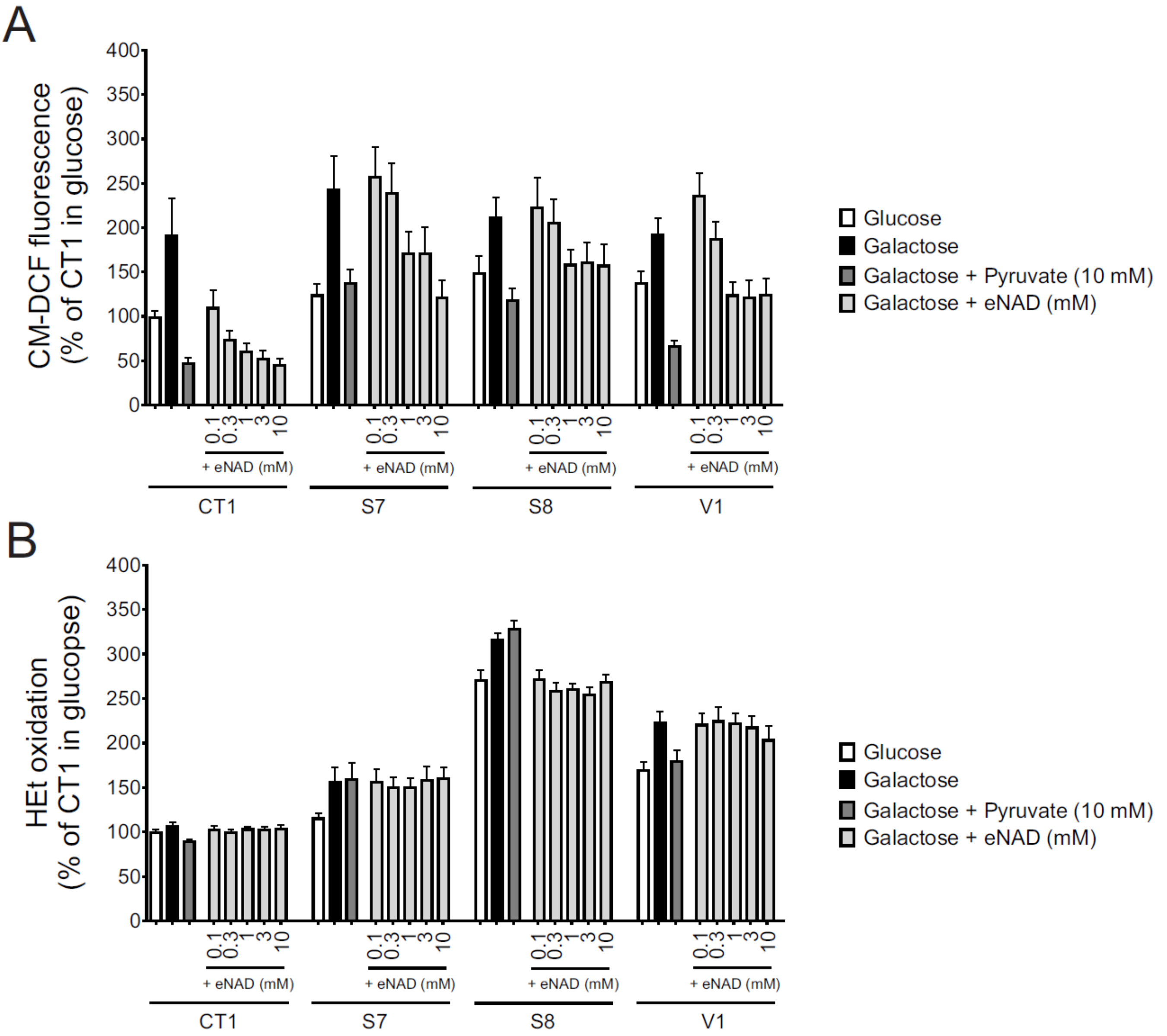

Supplementary figure S12: Data for individual cell lines (CT1, S7, S8, V1) used to create figure 6A and 6B. See Results for details.

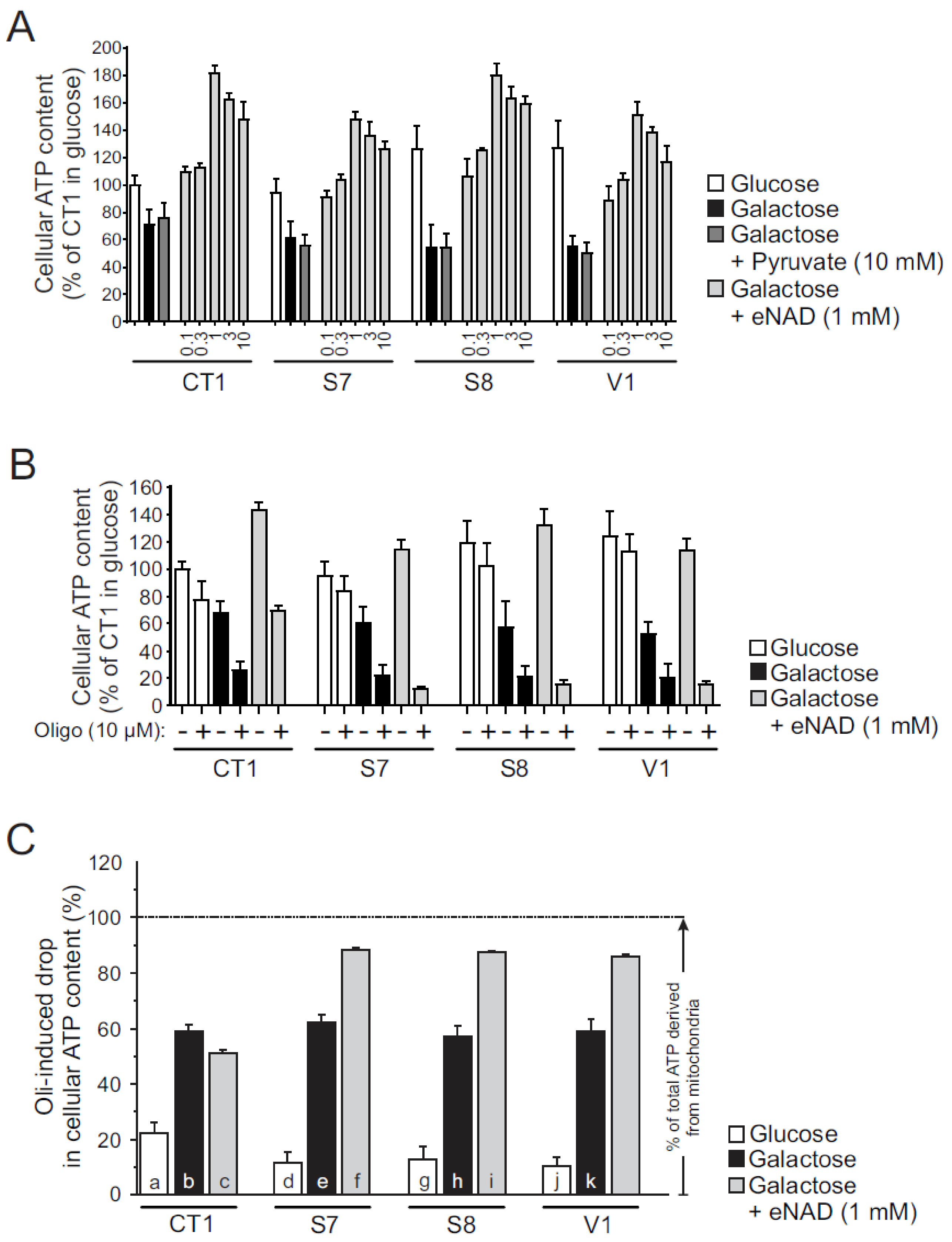

Supplementary figure S13: Data for individual cell lines (CT1, S7, S8, V1) used to create figure 7A, B and C. See Results for details
